# Asymptotic theory of time-varying social networks with heterogeneous activity and tie allocation

**DOI:** 10.1038/srep35724

**Published:** 2016-10-24

**Authors:** Enrico Ubaldi, Nicola Perra, Márton Karsai, Alessandro Vezzani, Raffaella Burioni, Alessandro Vespignani

**Affiliations:** 1 Institute for Scientific Interchange Foundation, 10126 Torino, Italy; 2 Dipartimento di Fisica e Scienza della Terra, Università di Parma, Parco Area delle Scienze 7/A, 43124 Parma, Italy; 3 INFN, Gruppo Collegato di Parma, Parco Area delle Scienze 7/A, 43124 Parma, Italy; 4 Centre for Business Network Analysis, University of Greenwich, Park Row, London SE10 9LS, United Kingdom; 5 Laboratory for the Modeling of Biological and Socio-technical Systems, Northeastern University, Boston MA 02115 USA; 6 Univ Lyon, ENS de Lyon, Inria, CNRS, UCB Lyon 1, LIP UMR 5668, IXXI, F-69342, Lyon, France; 7 Centro S3, CNR-Istituto di Nanoscienze, Via Campi 213A, 41125 Modena Italy

## Abstract

The dynamic of social networks is driven by the interplay between diverse mechanisms that still challenge our theoretical and modelling efforts. Amongst them, two are known to play a central role in shaping the networks evolution, namely the heterogeneous propensity of individuals to i) be socially active and ii) establish a new social relationships with their alters. Here, we empirically characterise these two mechanisms in seven real networks describing temporal human interactions in three different settings: scientific collaborations, Twitter mentions, and mobile phone calls. We find that the individuals’ social activity and their strategy in choosing ties where to allocate their social interactions can be quantitatively described and encoded in a simple stochastic network modelling framework. The Master Equation of the model can be solved in the asymptotic limit. The analytical solutions provide an explicit description of both the system dynamic and the dynamical scaling laws characterising crucial aspects about the evolution of the networks. The analytical predictions match with accuracy the empirical observations, thus validating the theoretical approach. Our results provide a rigorous dynamical system framework that can be extended to include other processes shaping social dynamics and to generate data driven predictions for the asymptotic behaviour of social networks.

Unveiling the mechanisms shaping the evolution of social networks is a challenging task that has captured the attention of researchers since many decades. Nowadays, the availability of large, high quality and time-resolved datasets is providing unparalleled opportunities to deepen our understanding of social dynamics^1^,^2^,^3^,^4^,^5^. A close inspection of such datasets shows that ties between individuals continuously change in time, and their dynamics are driven by different mechanisms that generate complex topological and dynamical features^5^,^6^,^7^,^8^,^9^,^10^.

The establishment of social ties is costly^11^,^12^,^13^,^14^, but associated to benefits^15^ for single actors and groups as it allows to access and gain social capital^16^,^17^. In the development and maintenance of social interactions, individuals *invest* heterogeneously according to diverse strategies. Firstly, not all individuals are equally socially active^4^,^18^,^19^,^20^,^21^,^22^. Indeed, people show different propensity to social interactions, resulting in a diverse number of contacts in a given observation time^18^,^23^. Secondly, individuals may allocate social interactions in different ways, either by favouring the strengthening of a limited number of strong ties (bonding capital) or by the exploration of weak ties opening access to new information and communities (bridging capital)^24^,^25^,^26^,^27^,^28^,^29^. Interestingly, recent works showed that these traditional findings apply not only to real world social-networks but also in workplaces^30^,^31^, online social networks^32^,^33^,^34^ and collaboration networks^35^,^36^,^37^,^38^. The origins of such heterogeneities are rooted in the trade off between competing factors, such as the need for close relationships^15^, the efforts required to keep social ties^11^, and temporal and cognitive constraints^12^,^13^,^14^. These mechanisms have long been acknowledged as key factors in the description of social networks^39^,^40^,^41^. In particular, they have been shown to affect their dynamical features^4^,^23^,^40^,^42^,^43^,^44^,^45^,^46^,^47^, and dynamical processes unfolding on their fabric^4^,^39^,^40^,^41^,^48^,^49^,^50^,^51^,^52^,^53^,^54^.

Here, we propose a dynamic network model, that generalises the recent modelling scheme of *activity-driven-networks*^23^,^50^,^55^. We introduce a rule of links formation^39^,^41^,^56^,^57^,^58^ that explicitly takes into account heterogeneity in social activity and tie allocation. In particular, we propose a general functional form for the social allocation mechanism, able to fit empirical observations in seven time-resolved datasets, describing three different types of social interactions: scientific collaborations, Twitter mentions, and mobile phone calls. We provide a thorough statistical characterisation of activity and formation of social ties in each network and we identify the basic parameters defining the dynamics of ties’ evolution. Interestingly, we observe, across all the datasets, that, the larger the degree, the smaller is the probability of creating a new tie.

Prompted by this statistical analysis, we study the Master Equation (ME) that describes the evolution of the network connectivity structure of the proposed model. We solve the ME in the asymptotic regime (large network size and long time evolution) thus formally defining the asymptotic form of the degree distribution as well as the scaling relations for degree, activity, and the functions characterising the tie formation mechanism. The analytical solutions capture very well the empirical behaviour measured in all the analysed datasets. Furthermore, they connect explicitly the evolution of social networks to the parameter regulating the emergence of social ties. The proposed analytical framework is general and can be solved for statistically different activity patterns. The presented results have the potential to pave the way for a general asymptotic theory of the dynamics of social networks by progressively integrating further social mechanisms.

## Results

We analyse seven datasets containing time-stamped information about three different types of social interactions: scientific collaborations, Twitter mentions, and mobile phone calls (see the Methods section for the details). We considered five scientific collaborations networks obtained from five different journals (*PRA*, *PRB*, *PRD*, *PRE*, and *PRL*) of the American Physical Society (APS), one Twitter mentions network (*TMN*), and one mobile phone network (*MPN*). We represent all datasets as time-varying networks, where each node describes an individual and each time-resolved link describes a social interaction. The nature of connections is different according to the specific dataset. Links might represent a collaboration resulting in a scientific publication, a Twitter mention, or a mobile phone call.

**Figure 1:**
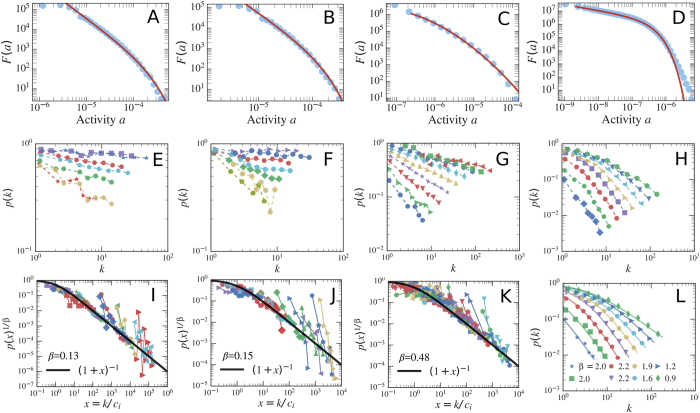
(**A**–**D**) The activity distribution *F*(*a*) for PRB (**A**), PRL (**B**), TMN (**C**) and the MPN (**D**) dataset. The solid lines represent the fit *F*(*a*) with the best functional form for each dataset. The latter are a truncated power law for the PRB, PRL and MPN case, while we find a lognormal for the TWT case (see Methods and SI Section 2 for details). In these plots we show the data starting from the lower bound of the fit, including all the statistically significant bins. (**E**–**H**) The measured *p*_*b*_(*k*) curves for selected nodes classes belonging to the PRB (**E**), PRL (**F**), TMN (**G**) and MPN (**H**) datasets. Each data sequence (different colours and markers) corresponds to a selected nodes class of the system, with the average activity of the class increasing from the lower to the upper curves. As one can see different nodes classes feature a differently behaving attachment rate function *p*_*b*_(*k*): for some nodes the probability to attach to a new node quickly drops to 0 at degree ≲10 while for some others the attachment probability is still ≳0.1 even at very large degree (*k* ~ 10^2^). (**I**–**K**) We rescale the attachment rate curves of all the nodes classes of the PRB (**I**), PRL (**J**) and TMN (**K**) datasets by sending *k* → *x*_*b*_ = *k*/*c*_*b*_ and then plotting the *p*_*b*_(*x*_*b*_)^1/*β*^, where *β* has the same value for every curve (see legends for the *β* values). For the MPN dataset (**L**) we show the original *p*_*b*_(*k*) curves belonging to a single nodes class with their fit. The resulting values of *β*_*b*_ are shown in the legend. The latter are found to fall in the 1.0 ≲ *β*_*i*_ ≲ 2.5 range.

In order to characterise the time-varying properties of such networks, we first measure the average activity *a*_*i*_. Formally, *a*_*i*_ is defined as the fraction of interactions of node *i* per unit of time and can be easily evaluated by counting the number of interaction engaged by the node *i* in the whole dataset. This quantity describes the propensity of nodes *i* to be involved in social interactions. Empirical measurements in a wide set of social networks show broad distributions of activity^23^,^31^,^41^,^50^,^51^. As shown in Fig. 1[A–D], we confirm these observations in our datasets. In particular, we find that in the APS and MPN datasets the activity is well fitted by a truncated power law, while in the TMN we find a Log-Normal distribution. We refer the reader to the Methods and Supplementary Information (SI) Section 1 for the details.

### Ties allocation

The activity *a*_*i*_ sets the clock for the activation of each node. However, when active, nodes might allocate their ties either exploring new connections or reinforcing already established one^58^. In order to characterise the mechanism regulating the establishment of ties, we group nodes in classes, labeled *b*, considering their activity *a* and final degree *k* (i.e. the cumulative number of distinct individuals contacted by each node at the end of the observation period). In other words, we assign nodes to classes *b* containing actors with statistically equivalent characteristics, i.e. nodes that engaged a similar number of interactions and that feature a comparable cumulative degree in the observation period (see SI for details). For each bin (class) *b*, we measure the probability *p*_*b*_(*k*) that, the next social act for nodes that have already contacted *k* distinct individuals, will result in the establishment of a new, *k* + 1-th, tie. The *p*_*b*_(*k*) function describes the social tie allocation process quantifying the inclination for a node to establish (when active) a new connection instead of re-connecting to an already contacted alter thus reinforcing a previous link. As shown in Fig. 1[E–H] *p*_*b*_(*k*) is, in general, a decreasing function of *k*. This observation resonates with previous research and empirical findings suggesting that the number of social interactions of an ego is bounded by cognitive and temporal constrains^11^,^12^,^13^,^14^. Indeed, the larger the number of alters in a social circle, the smaller the probability that the next social act will be towards a new tie.

The empirical findings suggest that the mechanism governing the allocation of social ties follow a general form that can be written as:


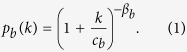


In this expression, the *β*_*b*_ modulates the tendency to explore new connections, while the *c*_*b*_ defines the intrinsic characteristic limit of the individual to maintain multiple ties. Although one could imagine more complicate analytical forms, we use this parsimonious approach to characterise the different datasets. Interestingly, we find that in the five co-authorship networks and Twitter, the exponent *β* is the same regardless of the class *b*. Furthermore, the values of *c*_*b*_ are typically peaked around a well defined value (see SI for details). More precisely, we can rescale the proposed functional form in each class *b* by defining the variable *x*_*b*_ = *k*/*c*_*b*_, yielding


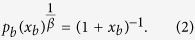


In the presence of a single exponent *β* characterising the system, as shown in Fig. 1[I–K], all empirical curves do collapse on the reference function (1 + *x*)^−1^. The data collapse however is not occurring in the case of the MPN dataset. In the latter we find a more heterogeneous scenario in which different nodes’ classes are characterised by different values of *β*_*b*_ and *c*_*b*_, see Fig. 1L. Note that in Fig. 1[I–K] some curves deviate from the reference function due to a poor statistics of the points (i.e. a small number of measures of the *p*_*b*_(*k*) for each value of *k*). We do not show the *p*_*b*_(*k*) estimation error for clarity. Nevertheless, these fluctuations are marginal and due to a limited amount of events. In fact, the proposed functional form captures the *p*_*b*_(*k*) behaviour for the systems’ bulk and allows us to develop a model able to correctly predict the network evolution.

**Figure 2:**
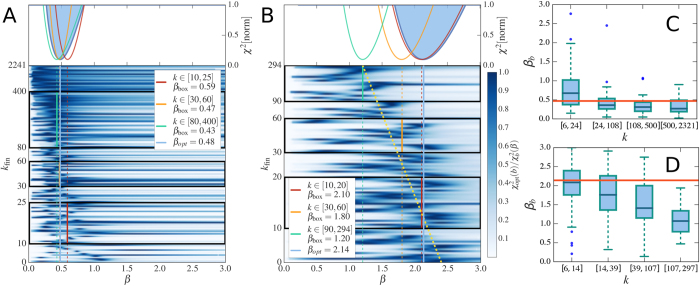
The heat-map-like quality of fit 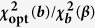 (bottom plots, see SI for details). We show the quality of fit as a function of the exponent *β* (*x*-axes) and different nodes bins *b* sorted by their final degree (*y*-axes) for (**A**) the TMN and (**B**) the MPN datasets, respectively. The color-map (see colorbar for values) is proportional to 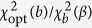, being 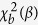 the sum of the squared residuals of bin *b* at a given *β* and 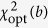 the minimum value of this sum for bin *b*. This ratio represents the goodness of fit: the darker, the higher. The cyan vertical line is the optimal value of *β*, *β*_*opt*_, that gives the best overall fit of the *p*_*b*_(*k*) reinforcement function (see SI for details), while the other vertical lines represent the same quantity evaluated in the three black boxes corresponding to different final degree intervals. (Top plots) The overall goodness of fit *χ*^2^(*β*) (up-filled curve) and the same quantity for the three final degree intervals (see Supplementary Information for details). For (**A**) Twitter we find a single value of *β*_*opt*_ = 0.48 to fit most of the curves, while in the MPN case (**B**) a single *β*_*opt*_ = 2.14 does not catch all the curves. We also show a “guide-to-the-eye” to highlight this feature (yellow dashed line). In the box-plots on the right we show the distribution of the optimal *β* for each nodes bin *b* for the TMN dataset (**C**) and the MPN one (**D**), having grouped the bins accordingly to their final degree *k*. The boxes extend from the lower to the upper quartile, while whiskers show the range of data (outliers are shown as blue points). We also plot the median of each bins group (blue lines inside the boxes) and the overall optimal exponent *β*_opt_ (solid orange lines).

The different behavior of *p*_*b*_(*k*) between the paradigmatic cases of TMN and MPN is explained in Fig. 2A,B. The bottom plots show the quality of fit for each value of *β* and for each bin *b* (the bluer, the better). In particular the scale of blue tones represents 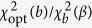, where 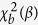 is the sum of the squared normalized residuals found when fitting the empirical *p*_*b*_(*k*) curve with Eq. (1) (in the fitting procedure we keep fixed *β*_*b*_ = *β* and we optimize over *c*_*b*_), while 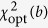 is the minimum of 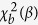 as a function of *β* in the bin *b*.

Figure 2A, shows a single value of *β*, 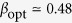, to fit most of the curves in the TMN dataset (*β*_opt_ is the best overall fit of the reinforcement function, see SI for details). On the other hand, in Fig. 2B we find a different behavior for the MPN dataset: there is not a single value of *β* fitting most of the bins. Indeed, as we increase the final degree of the bins, the value *β*_*b*_ giving the best fit for each bin *b* lowers. This difference is evidenced in the upper plots where we plot the residuals as computed for different sub-sets of nodes bins selected in the system. In the MPN dataset the best fits fall in a wide range of *β* values, from 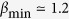 to 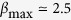. To further support this result we also show in Fig. 2C,D the box-plots of the optimal values of *β*_*b*_ for each bin *b*. Specifically, we divide all the bins in four groups depending on their final degree and display the distribution of the optimal *β*_*b*_ for each group of bins. In the TMN dataset (Fig. 2C) *β*_opt_ = 0.48 fall in the lower-upper quartiles range for all the groups. On the other hand, in the MPN dataset (Fig. 2D) in the high degree bins *β*_opt_ does not fall in the lower-upper quartile interval. Moreover, in the last bin subset *β*_opt_ falls outside the range of *β*_*b*_ values. In the SI we provide further details on the fitting procedure.

### Dynamic network model

By leveraging on the empirical evidence gathered, it is possible to define a basic generative model of network formation based on two stochastic mechanisms. Let us define the network 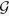 containing *N* nodes. At each time step a node *i* is active according to a probability *a*_*i*_ drawn from distribution *F*(*a*)^23^,^31^,^41^,^50^,^51^. Once active, the node *i* (assuming that has already contacted *k* different agents) will contact a new, randomly chosen node with probability 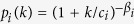. Otherwise, with probability 1 − *p*_*i*_(*k*), it will interact with an already contacted node chosen at random among its neighbours. Interactions are considered to last one single time step. For this model it is possible to write explicitly the Master Equation (ME) describing the evolution of the probability distribution *P*_*i*_(*k*, *t*) that a node *i* has degree *k* at time *t*:


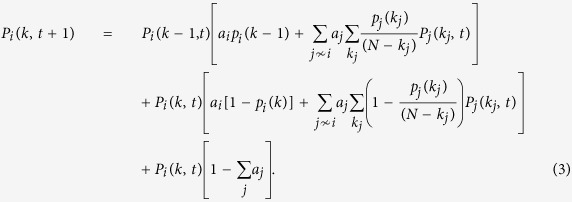


In the above equation the sums in *j* ~ *i* and 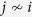 run over the nodes already contacted and not yet contacted by *i*, respectively. *k*_*j*_ describes the degree of each node *j*. Moreover, we work in the *a* ≪ 1 approximation, so that we assume that only one node is active for each evolution step. The first two terms on the right hand side of (3) account for the increment of the number of nodes having degree *k* − 1. The former occurs when node *i* having degree *k* − 1 gets active and contacts a new node with probability *a*_*i*_*p*_*i*_(*k* − 1). On the other hand the latter one is effective when node *i* gets contacted by node *j* of degree *k*_*j*_ (that never got in contact with *i* before) that activates and attaches to node *i* with probability *a*_*j*_*p*_*j*_(*k*_*j*_)/(*N* − *k*_*j*_). In the latter, the 1/(*N* − *k*_*j*_) factor accounts for the probability of *j* to exactly select node *i* amongst the *N* − *k*_*j*_ nodes outside of the *j*'s neighbourhood of size *k*_*j*_. Likewise, the third and fourth terms of the r.h.s. of the equation account for the conservation of the number of nodes of degree *k*. This is achieved either when node *i* gets active and contacts one of its neighbours with probability *a*_*i*_(1 − *p*_*i*_(*k*)), or when *i* gets contacted by one of its neighbours. The last line of Eq. (3) accounts for the possibility in which no node gets active in the current evolution time step, thus conserving the *P*_*i*_(*k*, *t*). Given the *a* ≪ 1 approximation this term reads 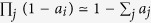.

### Asymptotic theory for networks with *β*_*b*_ = *β*

Let us focus on networks characterized by a single exponent *β*. We will consider the large time and degree 1 ≪ *k* ≪ *N* limits, so that *k* can be approximated by a continuous variable and *N* − *k* ≈ *N*. Clearly, we expect that such a regime holds for large time but still far from the *k* ~ *N* saturation regime. By neglecting the sub-leading terms of order 1\*t* we can thus write the continuous asymptotic version of Eq. (3) as


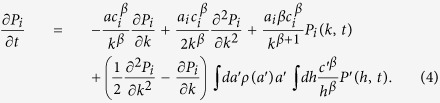


This equation can be solved explicitly (see SI for details), yielding the asymptotic form:


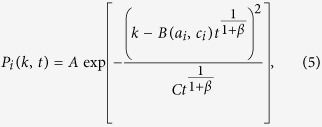


where *A* is a normalisation constant, *C* a constant and *B*(*a*_*i*_, *c*_*i*_) a multiplicative factor of the *t*^1/(1+*β*)^ term that depends on the activity *a*_*i*_ and *c*_*i*_ of the considered agent. Its implicit expression is given in the SI, however 
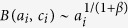
 for large *a*_*i*_.

A first general result concerns the evolution in time of the average degree 〈*k*(*a*, *t*)〉 of nodes belonging to a given activity class (where we dropped the *i* index) that follows the scaling laws


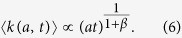


**Figure 3:**
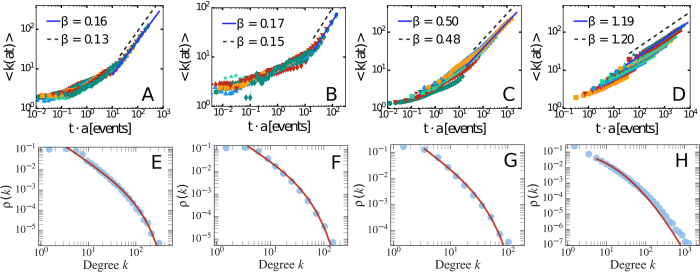
(**A**–**D**) The rescaled 〈*k*(*at*)〉 curves for selected nodes classes belonging to the PRB (**A**), PRL (**B**), TWT (**C**) and MPN (**D**) datasets. The time of the original data (symbols) is rescaled with the activity value *t* → *at*. We also show the fitting curve 〈*k*(*t*)〉 ∝ *t*^1/1+*β*^ (blue solid lines) and the expected asymptotic behaviour (black dashed lines). In the MPN case (**D**) we fit using *β* = *β*_min_ = 1.2. (**E**–**H**) The degree distribution *ρ*(*k*) for the PRB (**E**), PRL (**F**), PRA (**G**) and TMN (**H**) datasets. The predicted functional form of *ρ*(*k*) found in Eq. (7) and Table (1) is shown for comparison (red solid lines). As in Fig. 1 we show the data starting from the lower bound of the degree distribution, including in the plot all the statistically significant measures of the probability density function *ρ*(*k*).

The growth of the system is thus modulated by the parameter *β* that sets the strength of the reinforcement of ties. In the limit case *β* = 0 the growth would be linear. Indeed, the reinforcement of previously activated ties would be zero and nodes would keep connecting randomly to other vertexes, thus increasing their social circle. In the opposite limit *β* → ∞ each node would create, and constantly reinforce, just one tie, i.e. the first established. In the six datasets described by a single *β* value, we observe the range 0.13 ≤ *β* ≤ 0.48 that indicates a sub-linear growth of the social system. In Fig. 3 we find a very good agreement between the analytical prediction of Eq. (6) and the empirical 〈*k*(*a*, *t*)〉 curves, obtaining the first empirical validation of the modelling framework proposed and its ability at capturing the network formation dynamics.

Furthermore, Eq. (6) connects, at a given time *t*, the degree *k* and the activity *a* of a given node, as 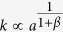. Thus, given any specific activity distribution *F*(*a*), we can infer the functional form of the degree distribution *ρ*(*k*) by substituting *a* → *k*^1+*β*^, finding:


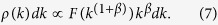


It is important stressing that the analytical framework is not limited to a specific functional form of the activity. Indeed, with an arbitrary functional form of *F*(*a*), Eq. (6) gives us the possibility to predict the behaviour and parameters of the corresponding degree distribution. In Table 1 we report the degree distribution predicted by Eq. (6) for activities following a common set of heavy-tailed distributions, i.e. power-laws, truncated power-laws, stretched exponentials, and log-normal, that are usually found in empirical data. In Fig. 3[E–G] we compare the degree distributions *ρ*(*k*) predicted by Eq. (7) with real data. Interestingly, also in this case the functional form obtained from the analytical solutions of the model fits remarkably well the empirical evidence. It is important to notice that *ρ*(*k*) is also function of the parameter *β*. In other words, the connectivity patterns emerging from social interactions can be inferred knowing the propensity of individuals to be involved in social acts, the activity, and the strength of the reinforcement towards previously establish ties, *β*. Finally it is worth remarking that Eqs (6 and 7) are not affected by the distribution of *c*_*i*_. This is an important result as it reduces the number of relevant parameters necessary to define the temporal evolution of the system.

### Asymptotic theory for networks with distributed *β*

As we already mentioned, in the *MPN* dataset we find the evolution of social ties described by a distribution of *β* rather than a single value of it. This observation points to a more heterogeneous distribution of social attitudes with respect to the other six datasets analyzed. Arguably, such tendency might be driven by the different functions phone calls serve enabling us to communicate with relatives, friends or rather to companies, clients etc. The need to introduce different values of *β* in the system complicates the model beyond analytical tractability (see SI for details). Nevertheless, we find that the leading term of the evolving average degree can be described by introducing a simplified model. In this, nodes feature different values of *β* and undergo a simplified dynamics (see the Methods section and the SI for details) that neglects, for every node, the effects of links established by others. In these settings, we can solve the ME and show that the minimum value of *β*, *β*_min_, rules the leading term of the evolving average degree. In other words, we find that even in this case 〈*k*(*a*, *t*)〉 evolves as in Eq. (6) but with *β* substituted by *β*_min_. As shown in Fig. 3D the analytical predictions coming from the simplified model find good agreement with empirical evidences of *MPN* dataset where 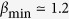 as evidenced in in Fig. 2. It is interesting to notice that the nodes characterized by *β*_min_ are those with the weak tendency to reinforce already established social ties. They are social explorers^58^. Notably, our results, indicate that they lead the growth of average connectivity of the network.

## Discussion

The empirical finding presented here shows clearly that the “cost” associated to the establishment of a new social tie (i.e. the 1 − *p*(*k*) probability to contact an already contacted node instead of a new one) is not constant but function of the number of already activated connections. This supports the idea that social capabilities are limited by cognitive, temporal or other forms of constraints^12^,^13^,^14^. By framing such empirical finding in a simple stochastic model of network formation, we derived a general asymptotic theory of network dynamics and extracted the general scaling laws for the behaviour in time of average degree and degree distribution.

This work introduces some advances in the fields of time-varying networks and more in general in social network modelling. It proposes a thorough measure and characterisation of social tie allocation and defines a general functional form for this process that fits seven datasets describing three different types of social acts.

Furthermore, the proposed network model can be treated analytically. In particular, the asymptotic solutions correctly predicts the growth of average degree, and also provide new insights on social dynamics by explicitly connecting individuals’ activity and degree^58^. Remarkably, this connection does not depend on the functional form of the activity distribution so that the analytical results are valid for general activity patterns. Moreover, the model allows for the implementation of different functional form of the reinforcement mechanism *p*(*k*) and can be easily extended to account for more complex rules governing the evolution and emergence of social interactions.

The model represents a good test-bed for the study and characterisation of dynamical processes unfolding on top time-varying networks. Indeed, it couples two mechanisms that have been shown to deeply affect the outcome of dynamical processes: nodes’ activation^23^,^50^,^59^ and social tie allocation^41^,^60^,^61^.

The model comes with some shortcomings. Indeed, it does not capture the modular structure or, more in general, correlations beyond the nearest neighbourhood that are typical of many social networks^62^. In fact, individuals tend to organise their social circles in tight, often hierarchical, communities. These features can be introduced by means of more refined mechanisms as illustrated in recent work^56^, but they do not affect the results presented here as we focus on local topological quantities. The model does not capture the burstiness typical of social acts^21^,^63^. We consider a simplified Poissonian scheme of nodes activation. A recent extension of the activity driven framework, without the reinforcement mechanism acting on social ties, has been proposed to account for non Poissonian node dynamics^64^. This is the natural starting point to generalise our model to bursty activities. Furthermore, the model does not consider the turnover of social ties^58^. Indeed, in our framework once a social connection has been established it cannot be eliminated in favour of others. Clearly, this feature is of particular importance when considering social systems evolving on longer time scales, as the scientific journals we studied here, and might influence the measurement of the parameters describing evolution of the ego-networks.

Notwithstanding these limitations, the modelling framework we proposed paves the way to a deeper understanding of the emergence and evolution of social ties. The agreement between the analytical predictions and observed behaviours in seven real datasets, describing different types of social interactions, are encouraging steps in this direction. Finally, our results are a starting point for the development of predictive tools able to forecast the growth and evolution of social systems based not just of regression models or simplified toy models but on a more rigorous analysis of ego-network dynamics.

## Methods

### Datasets

We analysed seven large-scale time resolved networks describing three different types of social interactions.

- Five networks from the APS datasets. These consider the co-authorship networks found in the Journals of the American Physical Society^65^. Specifically, the PRA dataset covers the period from Jan. 1970 to Dec. 2006 and contains 36,880 papers written by 34,093 authors and connected by 100,683 edges. The PRB dataset refers to the Jan. 1970 to Dec. 2007 period and contains 104,047 papers published by 84,367 authors which are connected by 416,048 links. The PRD datasets covers the same period as the PRB one and it is composed by 33,376 papers, 21,202 authors and 60,033 edges. The PRE dataset refers to the Jan. 1993 to Dec. 2006 period with 24,204 papers published by 28,188 authors connected by 68,029 edges. Finally, the PRL dataset contains all the 66,422 papers published between Jan. 1960 to Dec. 2006 and written by 78,763 authors forming 299,017 edges.
- One network dataset describing Twitter mentions (TMN), exchanged by users from January to September 2008. The network has 536,210 nodes performing about 160 M events and connected by 2.6 M edges.
- One Network dataset describing the mobile phone calls network (MPN) of 6,779,063 users of a single operator with about 20% market share in an undisclosed European country from January to July 2008. The datasets contains all the phone calls to and from company users thus including the calls towards or from 33,160,589 users in the country connected by 92,784,825 edges.

### Asymptotic solution of the ME for distributed *β*_*i*_ values

The solution of Eq. (4) found in Eq. (5) holds if the system feature a single value of *β*. As already discussed in the MPN dataset we find multiple values of *β* ranging from a minimum value, *β*_min_ to a maximum one *β*_max_. To find a prediction of the long time behaviour of such a system, let us propose a simplified model in which we focus on a single agent whose parameters are *a*_*i*_, *β*_*i*_ and *c*_*i*_. In this simplified version the agent can only call other nodes in the network, i.e. we neglect the contribution coming from the incoming calls). In this approximation we have to solve a modified version of Eq. (3), obtained by discarding all the terms containing the activity *a*_*j*_ of the nodes *j* ≠ *i*. By repeating the same procedure above, we get to the continuum limit that reads:


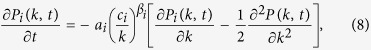


whose solution is similar to Eq. (5), the only differences being the value of *β* = *β*_*i*_ and the behaviour of the *B*(*a*_*i*_, *c*_*i*_) constant (see SI Section 3 for details). Interestingly, even in this case we find an average degree 〈*k*(*a*, *t*)〉 growing accordingly to the exponent *β*_*i*_, i.e. 
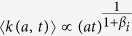
. Now, let us create a reservoir of *N* distinct nodes of equal activity *a* and assign to each of them a different value of *β*_*i*_ drawn from an arbitrary distribution *P*(*β*_*i*_). Let us also group these nodes in *B* classes, defined so that each class *i* contains all the nodes featuring a similar value of *β* ~ *β*_*i*_. If we now let these *N* nodes evolve following the simplified model above, the average degree of each class *i* will grow as 
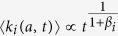
. Then, in the long time limit, the minimum value of *β*_*i*_, i.e. *β*_min_, will lead the growth of the ensemble’s average degree (see SI for further details), i.e.


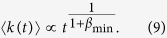


### *F*(*a*) and *ρ*(*k*) distributions from real data

We implement the method found in ref. 66 to determine the most likely functional form of both the activity and degree distributions. The fitting procedure is as follows: for each functional form of the distribution considered (power law, log-normal, truncated power law and stretched exponential) we first determine the *x*_min_ value, i.e. the lower bound to the functional form behaviour. The *x*_min_ value is defined as the value that minimises the Kolmgorov-Smirnov (KS) distance between the analytical complementary cumulative distribution (CDF) and the CDF of the data. The latter are found for each value of *x*_min_ by computing the optimal parameters of the distribution using the maximum-likelihood estimator (MLE). Then, comparing the CDF(*x* ≥ *x*_min_) of the data *S*(*x*) with the analytical one *S*(*x*), we compute the KS-distance as the maximum distance between the two CDF, i.e. 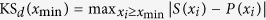. Once all the distances are computed we determine *x*_min_ as the values at which the minimum distance is recorded, i.e. *x*_min_ = min_*x*_KS_*d*_(*x*) (see SI and ref. 66 for details). Once we compute all the parameters for all the functional forms analysed we compare them with the *likelihood ratio test* 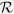 combined with the *p*-value that gives the statistical significance of 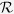 (see SI for details). The result of this procedure gives us the best candidate for the *F*(*a*) for each dataset. We find that a truncated power law is the best candidate for all the APS datasets together with the MPN one. The only exception is the TMN that displays a Log-Normal distribution of activity (see Figs 1 and SI for details). After we estimate the functional form and the parameters of the activity distribution *F*(*a*), Eq. (7) gives us the possibility to predict both the functional form of the degree distribution *ρ*(*k*) and the values of the parameters of such a distribution (e.g. the *α* exponent in a power-law with cutoff, see Table 1 for details). The degree distribution can then be fitted by optimising over the non-scale-free parameters for whose values we do not have an analytical or numerical prediction (e.g. the cut-off *τ* in a power-law with cutoff). Indeed, we are missing the value of the constant in front of the (*at*)^1/1+*β*^ term in the growth of the average degree 〈*k*(*a*, *t*)〉 in Eq. (6).

## Additional Information

**How to cite this article**: Ubaldi, E. *et al*. Asymptotic theory of time-varying social networks with heterogeneous activity and tie allocation. *Sci. Rep.* **6**, 35724; doi: 10.1038/srep35724 (2016).

## Supplementary Material

Supplementary Information

## Figures and Tables

**Table 1:** The functional form of the activity PDF *F*(*a*) and the predicted functional form of the *ρ*(*k*) degree distribution as found in Eq. (7), i.e. by replacing *a* → *k* ^1+*β*^.

PDF	*F*(*a*)	*ρ*(*k*)
Power Law	*a*^−*ν*^	*k*^−[(1+*β*)*ν*^ − ^*β*]^
Stret. Exp.	*a*^−*ν*−1^exp[−*λa*^*ν*^]	*k*^[(1+*β*)(*ν−*1)+*β*]^exp[−*τk*^(1+*β*)*ν*^]
Trunc. PL	*a*^−*ν*^exp[−*λa*]	*k*^−[(1+*β*)*ν−β*]^exp[−*τk*^(1+*β*)^]
Log-Normal	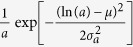	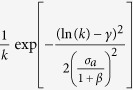

This substitution fixes the scale free parameters of the resulting distribution, i.e. the exponent of the power-law and of the *k* terms in the first three cases, and the STD 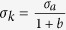 in the Log-Normal case. The free parameters over which we fit the degree distribution are: (i) the cut-off *τ* in the stretched exponential and power-law with cut-off and (ii) the *γ* mean value in the Log-Normal case. The selected PDF are, from top to bottom: power law, stretched exponential (Stret. Exp.), power law with cutoff (Trunc. PL) and the Log-Normal distribution.
