# Supplementary material for: Asymptotic theory of time-varying social networks with heterogeneous activity and tie allocation
Source: Sci Rep. 2016 Oct 24;6:35724. doi: 10.1038/srep35724 (PMC5075912; doi:10.1038/srep35724)
Supplement: Supplementary Information [file srep35724-s1.pdf]

Supplementary Information

Asymptotic theory of time-varying social networks with heterogeneous activity and tie allocation

Enrico Ubaldi[1,2,3], Nicola Perra[4,5], Márton Karsai[6], Alessandro Vezzani[2,7], Raffaella Burioni[2,3], and Alessandro Vespignani[1,5]

[1]Institute for Scientific Interchange Foundation, 10126 Torino, Italy
[2]Dipartimento di Fisica e Scienza della Terra, Università di Parma, Parco Area delle Scienze 7/A, 43124 Parma, Italy
[3]INFN, Gruppo Collegato di Parma, Parco Area delle Scienze 7/A, 43124 Parma, Italy
[4]Centre for Business Network Analysis, University of Greenwich, Park Row, London SE10 9LS, United Kingdom
[5]Laboratory for the Modeling of Biological and Socio-technical Systems, Northeastern University, Boston MA 02115 USA
[6]Laboratoire de l'Informatique du Parallélisme, INRIA-UMR 5668, IXXI, ENS de Lyon, 69364 Lyon, France
[7]Centro S3, CNR-Istituto di Nanoscienze, Via Campi 213A, 41125 Modena Italy

1 Data-sets

1.1 American Physical Society

The *APS* dataset contains the five co-authorship networks of five journals of the *A*merican *P*hysical *S*ociety, i.e., *Physical Review A*, *B*, *D*, *E* and Letters (*L*).

The various datasets contains the data referring to all the issues of the single journals from their first issue up to a certain edition, specifically:

- *PRA* from January 1970 to December 2006;
- *PRB* and *PRD* from January 1970 to December 2007;
- *PRE* from January 1993 to December 2006;
- *PRL* from February 1960 to December 2006.

Each dataset is composed by several files (one per month). Each file has as many lines as the number of papers published in that month. Finally, each line contains the IDs of the authors of the specific paper. For instance, the typical head of a file is:

```
Author_000    Author_001    Author_002  #First Paper with 3 authors
Author_003    Author_004                #Second Paper with 2 authors
   . . .         . . .         . . .               . . .
```

The data are cleaned so as to not take into account the papers with a single author.

When analyzing this dataset we define the user's activity a_i as the number of engaged collaborations (e.g. an author i that publish two papers, the first with 3 co-authors and the second with a single co-author, has activity $a_i = 4$).

We do not include large collaborations in our analysis (papers with more than ten authors). Details on the applied procedure to get the data and perform name disambiguation can be found in literature [1].

1.2 Twitter Mention Network

The dataset of *Twitter* is composed by 273 daily files covering the period between January the 1st to September the 30th2008. The dataset contains the so called *fire-hose*, i.e., all the $16,329,466$ citations done by all the $536,210$ users in the given period. The nodes in the network are connected via $2,620,764$ edges.

Each file contains the daily events with the structure:

```
Citer_ID_00    Cited_ID_00    # Event 0
Citer_ID_01    Cited_ID_01    # Event 1
Citer_ID_02    Cited_ID_02    # Event 2
    . . .          . . .          . . .
```

In this work we consider all the citations performed by all the users on the platform in the selected period, without discarding any of the collected event.

When analyzing this dataset we define the user's activity a_i as the number of citation made by i, i.e. the number of events actually engaged by the node i.

1.3 Mobile Phone Network

The dataset of the Mobile Phone Calls (MPC) is composed by a single file containing the $1,949,624,446$ time ordered events with 1 second resolution covering the period between January and July of 2008 for $6,779,063$ users of a single operator with 20% market share in an undisclosed European country.

The dataset contains all the events from and toward users of the company (so that even the calls from non-company users to company users and vice-versa are taken into account). As a result, we have $33,160,589$ nodes (of which $6,779,063$ are users of the selected company) that are connected via $92,784,825$ edges.

We split the huge list of events in 98 files (each of them containing more or less the same number of events) for computing convenience. Each file contains events with the structure:

```
Caller_ID    Called_ID    Company_Caller    Company_Called    # Event 0
Caller_ID    Called_ID    Company_Caller    Company_Called    # Event 1
Caller_ID    Called_ID    Company_Caller    Company_Called    # Event 2
   . . .        . . .          . . .              . . .            . . .
```

where `Company_Caller` and `Company_Called` are the value of the provider company of the called and caller nodes, respectively (e.g. the value is set to 1 if the node is a customer of our company, 0 otherwise).

When analyzing this dataset we define the user's activity a_i as the number of calls done by the node, i.e. the number of calls actually engaged by the node i.

2 Data analysis

2.1 Activity distribution and the nodes binning

For the datasets presented in Section 1 we first evaluate, for each node i, the total number u_i of events engaged by the node itself. For instance u_i is the number of calls made by the node i in the MPC dataset or the number of citations done by i in the *Twitter* dataset.

We then define the node activity a_i as the ratio between the i-th node's number of events and the total number of events observed in the dataset, i.e. $a_i = u_i/u_{\text{tot}}$ where $u_{\text{tot}} = \sum_j u_j$. Thus, a_i falls in the range $a_i \in [\epsilon, 1.0)$ with $\epsilon = \min_i(u_i)/u_{\text{tot}}$. We then introduce and compute the activity distribution $F(a)$. In Fig. 1 we show the resulting activity distribution for each analyzed dataset, while in Table (1) we show the best candidate functional form for the $F(a)$ distribution of each dataset. The latter is estimated using the methods found in [2].

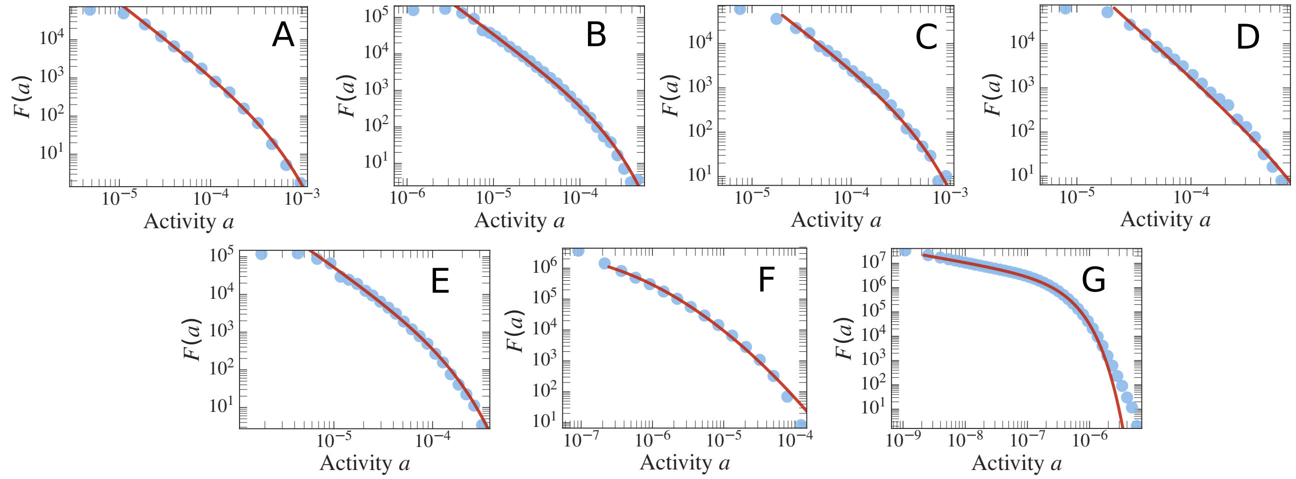

Fig. 1: The experimental activity distribution $F(a)$ for (A) PRA, (B) PRB, (C) PRD, (D) PRE, (E) PRL, (F) TMN and (G) MPN (blue points). We also show the best candidate fit of the $F(a)$ distribution (red solid lines) featuring the functional form and parameters found in Table (1). In all the plots we show the data and fit starting from the lower bound x_{min} of the distribution, including all the statistically-significant bins (i.e. the bins with at least 10 observations or equivalently with a relative error $\leq 30\%$ on the estimation). See Table (1) for the lower bound details.

Dataset	Distribution	Parameters	KS_d	%	$\mathcal{L}$
TMN	Lognormal	$a_{\min} = 4.28e-7$, $\mu = -14.31$, $\sigma = 1.83$	$1.5e-2$	52	$-2.26e+3$
PRA	Truncated	$a_{\min} = 1.90e-5$, $\lambda = 2.46e+3$, $\alpha = 1.85$	$1.5e-2$	43	-301
PRB	Truncated	$a_{\min} = 6.31e-6$, $\lambda = 6.07e+3$, $\alpha = 1.74$	$1.4e-2$	49	-744
PRD	Truncated	$a_{\min} = 4.54e-5$, $\lambda = 2.66e+3$, $\alpha = 1.67$	$1.6e-2$	37	-286
PRE	Truncated	$a_{\min} = 4.24e-5$, $\lambda = 1.23e+3$, $\alpha = 2.30$	$1.5e-2$	33	-264
PRL	Truncated	$a_{\min} = 1.10e-5$, $\lambda = 9.58e+3$, $\alpha = 1.80$	$1.7e-2$	39	-577
MPN	Truncated	$a_{\min} = 2.17e-9$, $\lambda = 3.82e+6$, $\alpha = 0.448$	$9.5e-3$	94	$-1.6e+4$

Tab. 1: The candidate functional form of the activity distribution for each analyzed dataset, the evaluated parameters (see Table [1] in the main for the analytical expressions), the Kolmgorov-Smirnov distance KS_d, the percent % of nodes in the dataset that have activity $a_i \geq a_{\min}$ and the normalized log-likelihood $\mathcal{L}$. In the parameters we include $a_{\min}$ that is the value of the activity that minimizes the KS distance. This is the lower bound for the functional form behavior, i.e. the point at which data behave as the functional form.

In particular, we compare the goodness of fit on the $F(a)$ distribution of the functional forms found in Table [1] of the main paper, i.e. power-law, truncated power-law, stretched exponential and log-normal distribution. The procedure for each dataset and each functional form reads as follows:

- we fit the $F(a)$ taking into account all the nodes featuring $a_i \geq x_{\min}$, where $x_{\min}$ is the lower bound of the distribution. The fit is performed using the maximum likelihood estimators (MLE) that return the optimal values of the parameters;

- once the optimal parameters are found we compute the Kolmogorov-Smirnov distance ($KD_d(x_{\min})$) between the analytical and experimental complementary cumulative distribution function (CDF);

- we then apply this procedure for different $x_{\min}$ and set the $a_{\min} = \min_{x_{\min}} KS_d(x_{\min})$ lower bound value as the one that minimizes the KS_d.

We then repeat this procedure for all the functional forms of the $F(a)$ and we then compare them with the *likelihood ratio test* $\mathcal{R}$ combined with the p-value that gives the statistical significance of $\mathcal{R}$ [2]. The result of this procedure gives us the best candidate for the $F(a)$ for each dataset as shown in Table (1). We find that a truncated power law is the best candidate for all the APS datasets together with the MPN one. On the other hand, in the TMN we find a log-normal distribution as the best candidate for the dataset.

Our datasets provide evidence that nodes within the same activity class (i.e. node with similar values of activity a_i) can feature very different memory behavior. In particular agents with large activity may connect to very few different nodes (strong reinforcement) or establish new links at almost every step (weak reinforcement). For this reasons each node i of the network is naturally classified according to her activity a_i and her final degree k_i, i.e. the total number of different agents that have been connected to i in the considered time window.

We then define a binning procedure that let us group together the similar nodes, i.e. nodes with similar activity and final degree. We divide the nodes in N_{act} activity classes so that within each activity class the most active node performs at most 1.5 times the events of the least active node. Then, with the same procedure, we further group the nodes within each activity class a according to their final degree, thus defining $N_{\text{deg}}(a)$ final degree classes. The nodes are therefore divided in $N_b = \sum_{a=1}^{N_{\text{act}}} N_{\text{deg}}(a)$ activity-degree classes. From now on, unless differently stated, whenever we mention the nodes' class or bin b we will be referring to one of these N_b classes.

2.2 The reinforcement process

To measure the reinforcement process of each system, we count all the communication events $e_b(k)$ engaged by every node i of the b-th class when it has degree $k_i = k$. In other words, $e_b(k)$ is the total number of events engaged by the nodes of the b-th class at degree k.

Each time an event engaged by a node i of the b-th class results in a degree increase $k_i = k \to k_i = k+1$, we increment the counter $n_b(k)$ by 1. In other words, $n_b(k)$ is the total number of events that the nodes belonging to the b-th and featuring degree k perform toward a new node. Of course, if a node i of the b-th class with degree $k_i = k$ increases its degree to $k_i = k+1$ because it gets called by a new node, the $n_b(k)$ counter is not incremented.

The best estimate of the probability for a new node to get establish a new connection at degree k then reads:

$$f_b(k) = \frac{n_b(k)}{e_b(k)}, \tag{1}$$

where $n_b(k)$ and $e_b(k)$ are the event counters as defined above. We can give an estimate of the uncertainty on $f_b(k)$, by assuming that at a given degree k the events are independent (i.e. there are no correlations between users) and by checking that $1 \ll n_b(k) \ll e_b(k)$ so that the STD $\sigma(f_b(k))$ of $f_b(k)$

reads:

$$\sigma(f_b(k)) = \sigma_b(k) = \sqrt{\frac{f_b(k)(1-f_b(k))}{e_b(k)}}. \tag{2}$$

We then fit $f_b(k)$ with the proposed reinforcement function $p_b(k,\beta)$:

$$p_b(k,\beta) = \left(1+\frac{k}{c(b)}\right)^{-\beta}, \tag{3}$$

where $c(b)$ is the social propensity of the b-th bin, k is the cumulative degree and β is the reinforcement strength, that will be kept fixed for all the nodes in the system. In particular, for each class b and with a fixed β, we optimize the parameter $c(b)$, by minimizing the function $\chi_b^2(\beta)$:

$$\chi_b^2(\beta) = \sum_{k=1}^{K_b} \frac{[f_b(k) - p_b(k,\beta)]^2}{\sigma_b(k)^2}, \tag{4}$$

where the index k runs over the K_b points of the b-th bin's curve and $\sigma_b(k)$ is as defined in Eq. (2). By repeating this procedure for each value of $\beta \in [0, 5.0]$ we find, for each class b, a $\chi_b^2(\beta)$ curve.

In Fig. 2 we show the behavior of $\chi_b^2(\beta)$. For each class b we find a minimum of $\chi_b^2(\beta)$, i.e. $\chi^2_{\mathrm{opt}}(b)$, at a certain $\beta_{opt}(b)$ (see the horizontal lines in the heat-map-like panels of Fig. 2).

Moreover, Fig. 2 shows that there are two different behaviors. Specifically, in the TMN case (see Fig. 2 (a)), one value of $\beta_{opt} = 0.48$ fits most of the curves, exception made for some outsiders: the value of $\beta_{\mathrm{opt}}(b)$ that maximizes the $1/\chi_b^2(\beta)$ is practically the same for all the bins. On the contrary, in the MPC case the maximum of the $1/\chi_b^2(\beta)$ function follows a diagonal path ranging from a larger $\beta_{\mathrm{opt}}(b)$ for bins with lower final degree to a smaller $\beta_{\mathrm{opt}}(b)$ for larger degree bins. In this case a single β_{opt} cannot fit all the curves and we have to consider a multi-β model where each class b features a different optimal value of β, $\beta_{\mathrm{opt}}(b)$.

In Fig. 3 we present the rescaled $p_b(k)$ curves for the PRA, PRD, PRE, PRL, TMN and MPC datasets. In the first five cases we show the rescaled curves obtained by substituting $k \to k/c_b$ and then plotting $p_b(k) \to p_b(k)^{1/\beta_{\mathrm{opt}}}$. As one can see, the curves nicely collapse on the reference curve $(1+k)^{-1}$. In the MPC case we show instead the original curves, each one fitted with its own $\beta_{\mathrm{opt}}(b)$. The latter parameter falls in the $1.2 \lesssim \beta_{\mathrm{opt}}(b) \lesssim 3.0$ interval for most of the curves as we also show in Fig. 2.

To quantitatively define the β_{opt} parameter, let us define the total mean square deviation $\chi^2(\beta)$ as

$$\chi^2(\beta) = \sum_{b=1}^{N_b} \left[\chi_b^2(\beta)\right], \tag{5}$$

where N_b is the total number of curves, i.e. the number of activity-degree bins b. Then, for the single exponent case, the function $\chi^2(\beta)$ allows to define β_{opt} as:

$$\beta_{\mathrm{opt}} = \min_\beta(\chi^2(\beta)). \tag{6}$$

In the multi-β case instead, we compute the different values of the exponent $\beta_{\mathrm{opt}}(b)$ found in the system by grouping the memory classes b accordingly to their final degree as shown in Fig. 2. The optimal value of $\beta_{\mathrm{opt}}(b)$ is found to be minimum for the bins featuring a large final degree, i.e. $\beta_{\min} \equiv \beta_{\mathrm{opt}} \sim 1.2$, which, as we will show in Section 3.2.3, is the exponent driving the evolution of the network.

To corroborate the results just outlined, we show in Fig. 4 the box plot of the $\beta_{\mathrm{opt}}(b)$ distribution for different groups of nodes classes b grouped by their final degree. We note that the APS and TWT datasets are well approximated by a single β_{opt} as the distribution of $\beta_{\mathrm{opt}}(b)$ within each sub-group of nodes is compatible with the global optimal value β_{opt}. On the other hand, in the MPN case we see that the large final-degree classes have their $\beta_{\mathrm{opt}}(b)$ distribution centered around a smaller value of

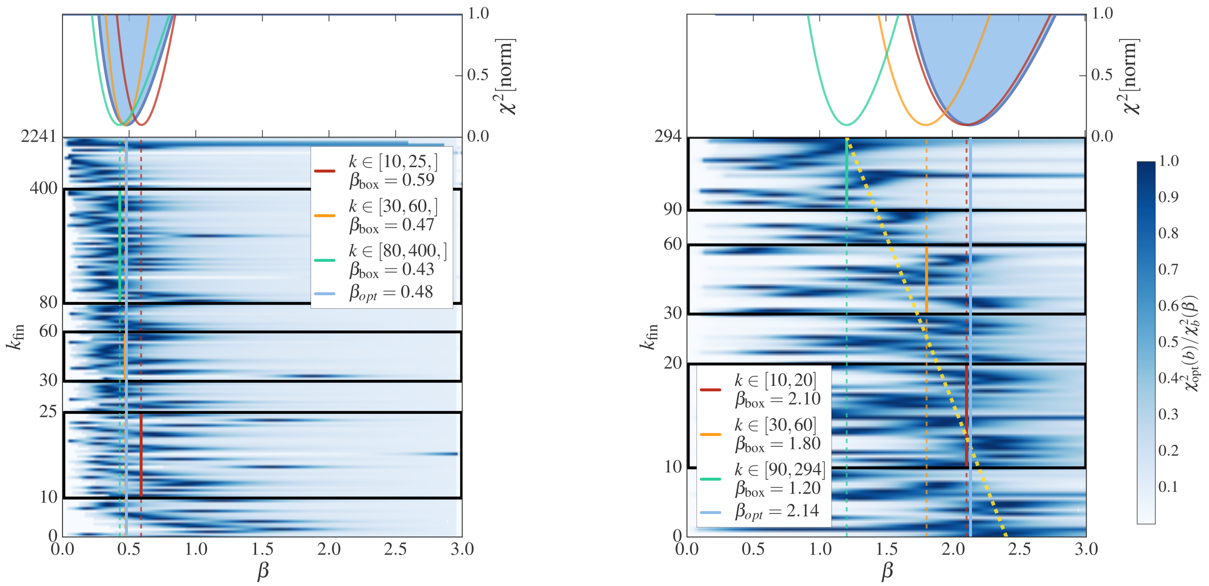

(a) (b)

Fig. 2: The heat-map-like value of $\chi^2_{\rm opt}(b)/\chi^2_b(\beta)$ (bottom plots). We plot the exponent β on the x-axes and the different bins b sorted by their final degree on the y-axes. The color-map is proportional to $\chi^2_{\rm opt}(b)/\chi^2_b(\beta)$ representing the goodness of fit: the darker, the higher. The cyan vertical line is the value of β_{opt} defined in Eq. (6), while the other vertical lines represent the same quantity evaluated in the three black boxes corresponding to different final degree intervals. (Top plots) The curve $\chi^2(\beta)$ as defined in Eq. (5) (up-filled curve) and the same quantity for the three final degree intervals. For Twitter (a): a single value of $\beta_{opt} = 0.48$ fits most of the curves and only some bins b deviate from the average behavior. (b) MPC: in this case we observe different behaviors depending on the final degree. Thus, a single $\beta_{opt} = 2.14$ does not fit all the curves. We also show a "guide-to-the-eye" to highlight this feature (yellow dashed line).

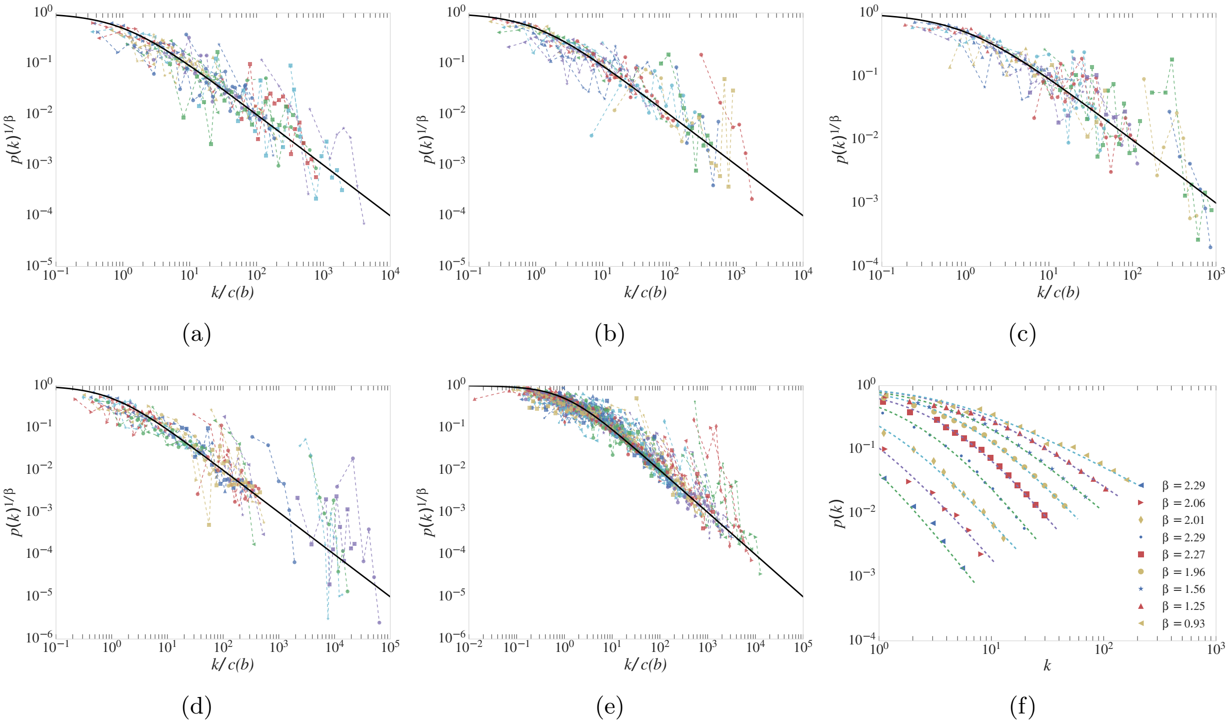

Fig. 3: Plot of the experimental $p_b(k)$ curves for the (a) PRA, (b) PRD, (c) PRE, (d) PRL, (e) TMN and (f) MPC datasets. In the (a-e) cases the k is rescaled as $k \to k/c(b)$, where $c(b)$ is the constant for the b-th class curve at $\beta = \beta_{\text{opt}}$. The $p_b(k/c(b))$ points are then rescaled sending $p_b(k/c(b))^{1/\beta_{\text{opt}}}$. In the (f) panel for MPC we simply plot $p_b(k)$ as a function of k with no rescaling, given that each curve features its own β optimal value β_{opt} as shown in the legend.

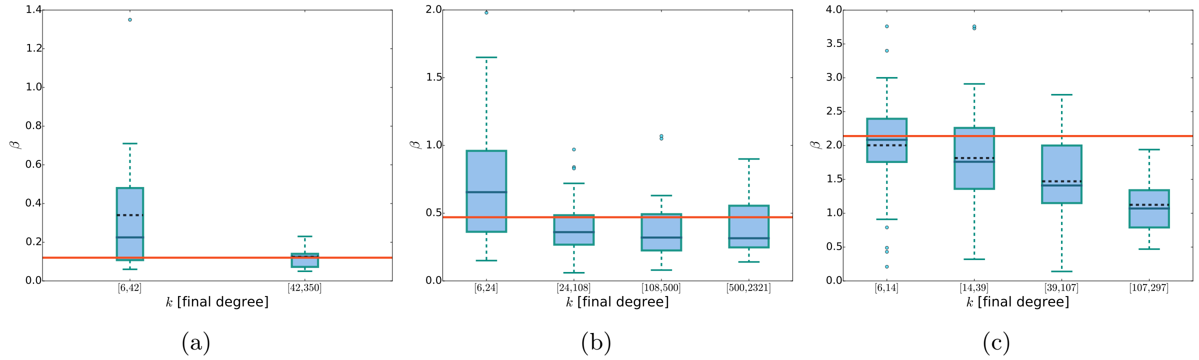

Fig. 4: The box plot representing the distribution for different range of nodes classes b of the $\beta_{\text{opt}}(b)$ for (a) PRB, (b) TMN and (c) MPN. We also show the global optimal value β_{opt} (horizontal red line) as found in Eq. (6). The height of the box corresponds to the lower and upper quartile values of the distribution and the horizontal solid line corresponds to the distributions median, while the dashed lines indicates the average value for each range of final degree. The whiskers extend from the box to values that are within $1.5x$ the quartile range. As one can see, in both the PRB and TMN datasets the optimal values β_{opt} is compatible with the distribution found in all the nodes class ranges (we find the same result for all the other APS datasets analyzed). On the other hand, in the MPN the distribution of $\beta_{\text{opt}}(b)$ lowers as the final degree of the class increases. The last group of nodes classes is no more compatible with the overall optimal β_{opt}, being the distribution centered around $\beta_{\text{opt}} \sim 1.1$, in agreement with our estimation of $\beta_{\text{min}} = 1.2$.

$\beta_{\min} \sim 1.2$. As already anticipated, this value will lead the asymptotic growth of the system as we will show in Section 3.2.3.

As a last remark we present in Fig. 5 (a, b) the measured distribution of the constant $c(b)$ for the MPN and TMN datasets. We show the distribution for all the nodes in the network and for each activity class a, i.e. the group of nodes featuring similar activity. The values of this constants are distributed but peaked around an average value. Moreover, the distribution of the $c(b)$ parameter within each activity closely follows the global one. The distribution of the social attitude $c(b)$ then appears to be a global, activity independent feature of the nodes in the system. Finally, in Fig. 5 (c) we show how the average value of the $c(b)$ constants, $\langle c\rangle = \langle c(b)\rangle_b$, differs from one dataset to the other varying from $\langle c\rangle = 0.8$ in PRB to $\langle c\rangle = 1.7$ in TMN and $\langle c\rangle = 4.6$ in the MPN case, respectively.

3 The model

3.1 Activity driven networks with no memory

The activity driven networks are an effective framework to describe time varying networks. The simplest memory-less model is defined as follows: the network consists of N nodes featuring an activity potential a_i, i.e. the probability for a node i to get active in a certain time interval dt reads $a_i dt$. The evolution rules are: *(i)* at each time step we start with N disconnected nodes; *(ii)* each node i whether gets active with probability $a_i dt$ or does not activate with probability $(1 - a_i)dt$. If a node gets active it calls a randomly selected node j in the network, thus creating an edge e_{ij}. *(iii)* At the end of the time step all the created connection are deleted and we start again from the initial step *(i)*.

These evolution rules define the Master Equation (ME) for $P_i(k, t)$, i.e. the probability that a node i of activity a_i has degree k at time t, where the degree k is the number of nodes that contacted i up to time t. We also set, without losing generality, $dt = 1$. The discrete time equation for $P_i(k, t)$ then reads:

$$P_i(k,t+1) = \tag{7}$$

$$a_i\frac{N-k}{N}P_i(k-1,t) + a_i\frac{k}{N}P_i(k,t) + P_i(k-1,t)\sum_{j\nsim i}a_j\sum_h\frac{P_j(h,t)}{N} +$$

$$P_i(k,t)\sum_{j\nsim i}a_j\sum_h P_j(h,t)\frac{N-1}{N} + P_i(k,t)\sum_{j\sim i}a_j + P_i(k,t)(1-\sum_j a_j). \tag{8}$$

The equation is obtained in the approximation where $a_i \ll 1$, so that between two consecutive times $t_i = t$ and $t_{i+1} = t + 1$ only one site can be active. We will assume that the activity a_i of a node i is small, i.e. $0 < a_i \ll 1$, and we will also consider the approximation $1 \ll k \ll N$ i.e. the integrated number of neighbors of a site is much larger than 1 but much smaller than the total number of agents N. The first term of the sum represents the probability that the site i is active and a new link is added to the system. The second term is the probability that the site i is active but this site connects to a site that has been already linked. In the third and fourth terms, the symbol $\sum_{j\nsim i}$ denotes the sum over the sites that are not yet connected to i. In particular, the third term represents the probability that one of these sites is active and that it connects to i. The fourth term is the probability that one of these sites is active but no link between j and i is established. The fifth term is the probability that one of the sites already connected to i is active (being $\sum_{j\sim i}$ the sum over the nodes already connected to i); in this case no new link is added to i. Finally, the last term represents the probability that at time t all the sites are not active. For $k \ll N$, the second term can be neglected. After some algebra we obtain the equation:

$$P_i(k,t+1) - P_i(k,t) = -\left(P_i(k,t) - P_i(k-1,t)\right)\left(a_i + \frac{1}{N}\sum_{j\nsim i}a_j\right)$$

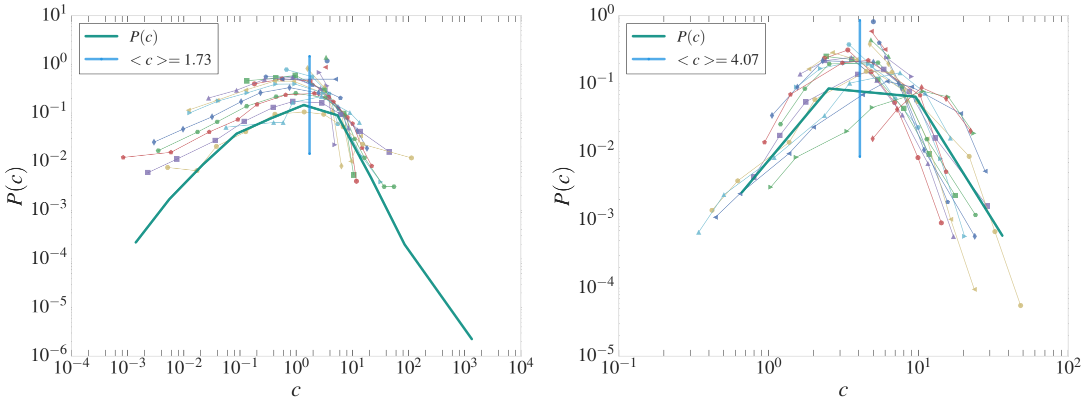

(a) (b)

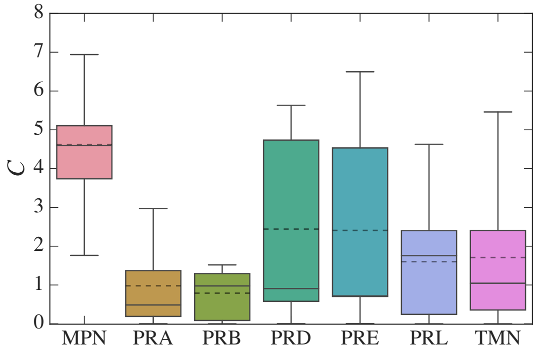

Dataset	β_{opt}	$\langle c(b) \rangle$
PRA	0.20	1.5
PRB	0.13	0.8
PRD	0.28	2.3
PRE	0.25	2.4
PRL	0.15	1.6
TMN	0.48	1.7
MPN	1.20	4.6

(c) (d)

Fig. 5: The $P(c)$ distribution of the constant $c(b)$ for the (a) TMN, and (b) MPN case (solid green line). We also compare the global $P(c)$ distribution with the distribution of the $c(b)$ values found within each activity class (solid lines and points): we find that the distribution of the $c(b)$ parameter is more or less activity independent as most of the distribution of the single activity classes follows the same functional form of the total distribution $P(c)$. We then report the average value $\langle c \rangle$ of the $c(b)$ constant for each dataset (vertical cyan line). The latter reads 1.71 for TMN and 4.62 for MPN. Note that in the single β case we evaluate $c(b)$ as the values of $c(b)$ that best fits the b-th $p_b(k, \beta)$ curve fixing β at its optimal value ($\beta = \beta_{opt}$). On the other hand, in the multi-β case we evaluate $c(b)$ as the ones that best fits the $p_b(k, \beta(b))$ curve, where the exponent is now foxed to the $\beta(b)$ value for the memory class b, i.e. to the optimal value for the class b. (c) The box plot showing the global distribution of the constant $c(b)$ in all the datasets analyzed. The height of the box corresponds to the lower and upper quartile values of the distribution and the horizontal solid line corresponds to the distributions median, while the dashed lines indicates the average value for each range of final degree. The whiskers extend from the box to values that are within $1.5x$ the quartile range. (d) In this table we report all the values of the reinforcement exponent β_{opt} and the average reinforcement constant $\langle c(b) \rangle$. For the MPN case we report the $\beta_{\text{opt}} = \beta_{\text{min}}$ and the constant values are evaluated for each nodes class b using its optimal value of β, $\beta_{\text{opt}}(b)$.

given that $\sum_h P_j(h,t) = 1$. For $k \ll N$, we assume that $\frac{1}{N}\sum_{j\nsim i} a_j = \langle a \rangle$ i.e. the average value of the activity. In the limit of large time and large k we can write a continuous equation in t and k obtaining:

$$\frac{\partial P_i(k,t)}{\partial t} = (a_i + \langle a \rangle)\left(-\frac{\partial P_i(k,t)}{\partial k} + \frac{\partial^2 P_i(k,t)}{\partial k^2}\right). \tag{9}$$

The solution of Eq. (9) is straightforward:

$$P_i(k,t) = (2\pi(a_i + \langle a \rangle)t)^{-\frac{1}{2}} \exp(-\frac{(k-(a_i+\langle a \rangle)t)^2}{2t(a_i+\langle a \rangle)}). \tag{10}$$

In the large time limit this solution reduces to a delta function: $P(a,k,t) = \delta(k-(a+\langle a \rangle)t)$. Therefore, the average degree $\langle k(a,t) \rangle$ of the nodes of activity a grows as:

$$\langle k(a,t) \rangle \propto (a + \langle a \rangle)t. \tag{11}$$

as already found in [3, 4]. Moreover the asymptotic degree distribution $\rho(k)$ of a network with activity distribution $F(a) \propto a^{-\nu}$ is:

$$\rho(k) \propto k^{-\nu}. \tag{12}$$

3.2 Plugging in the reinforcement process

The model presented in Sec. 3.1 is a basic model as it contains no correlations on an agent's story at all. In particular, the probability for a node i to re-call an already contacted node is independent of the node degree. While simple to describe and solve analytically, this model is not realistic, as there are no correlations in the each agent's history. Moreover, the probability to call an already contacted node is always small as $k/N \ll 1$ (and thus the probability to call a new node remains ~ 1 even at large degree k). However, as shown in Sec. 2.2, real-world systems features a strong reinforcement process, as the probability $p_i(k)$ to call a new node at degree k decreases as the degree k increases.

For this reason we introduce an extended version of the model described in [5] et al. which includes a reinforcement function $p_i(k)$ that measures the probability for an active node i, that has already contacted k different nodes in the network, to call a new node instead of an already contacted one.

3.2.1 The single β case

As already shown in Sec. 2.2 the functional form for the reinforcement process $p_i(k)$, i.e. the probability of adding a new link for the node i of degree k, reads:

$$p_i(k) = (1 + k/c_i)^{-\beta}. \tag{13}$$

By plugging Eq. (13) into Eq. (8) for node i, we get:

$$\begin{aligned} P_i(k,t+1) = P_i(k-1,t)\Big[a_i p_i(k-1) + \sum_{j\nsim i} a_j \sum_h \frac{p_j(h)}{(N-h)} P_j(h,t)\Big] + \\ P_i(k,t)\Big[a_i[1-p_i(k)] + \sum_{j\nsim i} a_j \sum_h \Big(1 - \frac{p_j(h)}{N-h} P_j(h,t)\Big)\Big] + \\ P_i(k,t)\Big[1 - \sum_j a_j\Big], \end{aligned} \tag{14}$$

where N is the number of nodes in the network, $\sum_{i\nsim j}$ is the sum over the nodes not yet connected to i and $\sum_j$ is the sum over all the N nodes of the network. Each term of Eq. (14) corresponds to a

particular event that may take place in the system, as already presented in the paper. For instance, the first term of the l.h.s. of Eq. (14) takes into account the increment of the node i's degree from $k-1$ to k. This may happen whether because node i gets active and contacts a new node in the system with probability $a_i p_i(k-1)$ or because a node j never contacted before gets active and calls exactly node i with probability $a_j p_j(h)/(N-h)$, being h the degree of j. In the same way, the second line takes into account that node i does not change degree k whether because it calls an already contacted node or because the non contacted nodes call other nodes in the network. The last line of Eq. (14) considers the possibility that no node in the network gets active.

If we now substitute Eq. (13) in Eq. (14), after some algebra we get:

$$P_i(k,t+1) - P_i(k,t) = \frac{a_i c_i^\beta}{(k-1+c_i)^\beta} P_i(k-1,t) - \frac{a_i c_i^\beta}{(k+c_i)^\beta} P_i(k,t) \\ - (P_i(k,t) - P_i(k-1,t)) \sum_{j \nsim i} a_j \sum_h \frac{P_j(h,t) c_j^\beta}{(N-h)(h+c_j)^\beta}. \quad (15)$$

Then, by applying the same approximations of large degree k and time t we obtain the continuous equation:

$$\frac{\partial P_i(k,t)}{\partial t} = - a \frac{c_i^\beta}{k^\beta} \frac{\partial P_i(k,t)}{\partial k} + \frac{a_i c_i^\beta}{2k^\beta} \frac{\partial^2 P_i(k,t)}{\partial k^2} + \frac{a_i \beta c_i^\beta}{k^{\beta+1}} P_i(k,t) + \\ \left(\frac{1}{2} \frac{\partial^2 P_i(k,t)}{\partial k^2} - \frac{\partial P_i(k,t)}{\partial k} \right) \int da_j F(a_j) a_j \int dc_j \rho(c_j|a_j) \int dh \frac{c_j^\beta}{h^\beta} P_j(h,t), \quad (16)$$

where $\rho(c_j|a_j)$ is the probability for a node j of activity a_j to have reinforcement constant c_j.

The long time asymptotic solution of Eq. (16) is of the form:

$$P_i(k,t) \propto \exp\left[-A \frac{(k - C(a_i,c_i) t^{\frac{1}{1+\beta}})^2}{t^{1/(1+\beta)}} \right], \quad (17)$$

Moreover, $C(a,c)$ is a constant depending on the activity a and the reinforcement constant c that follows the:

$$\frac{C(a,c)}{1+\beta} = \frac{ac^\beta}{C(a,c)^\beta} + \int da' F(a') \int dc' \rho(c',a') \frac{a' c'^\beta}{C(a',c')^\beta}. \quad (18)$$

We do not have an exact solution for $C(a,c)$, however $C(a,c) \simeq (ac^\beta)^{1/(1+\beta)}$ for large a.

Let us note that Eq. (17) can be obtained setting the variable $x = k - C(a) t^{\frac{1}{1+\beta}}$ and substituting it in Eq. (16) and imposing that $|x| \ll t^{\frac{1}{1+\beta}}$ from Eq. (16):

$$\frac{\partial P_i(x,t)}{\partial t} = \frac{a_i \beta c_i^\beta}{C(a_i,c_i)^{1+\beta} t} \left(x \frac{\partial P_i(x,t)}{\partial x} + P_i(x,t) \right) + \frac{C(a_i,c_i)}{2(1+\beta) t^{\frac{\beta}{1+\beta}}} \frac{\partial^2 P_i(x,t)}{\partial x^2} \\ - \frac{\partial P_i(x,t)}{\partial x} \int da_j F(a_j) \int dc_j \rho(c_j|a_j) \int dy \frac{a_j \beta c_j^\beta}{C(a_j,c_j)^{1+\beta} t} P_j(y,t) y. \quad (19)$$

The solution of the latter equation is of the form

$$P_i(x,t) \approx t^{-\frac{1}{2(1+\beta)}} \exp\left(-\frac{Ax^2}{t^{1/(1+\beta)}} \right) \quad (20)$$

thus confirming that x can be considered much smaller than $t^{\frac{1}{1+\beta}}$.

An important consequence of equations (17) and (18) is that, for a system featuring a reinforcement strength β, the average degree of the nodes belonging to a class b of activity a and constant c grows as:

$$\langle k(a,c,t)\rangle \propto C(a,c)\cdot t^{\frac{1}{1+\beta}}. \tag{21}$$

In particular, $\langle k(a,c,t)\rangle \propto (at)^{\frac{1}{1+\beta}}$ for large values of the activity a.

As expected, the average degree grows slower than in the memoryless case ($\beta = 0$) where the average degree grows linearly in time, as found in Eq. (11). Moreover, the presence of a reinforcement process also affects the asymptotic behavior of $\rho(k)$. Indeed, as already shown in the main paper, Eq. (21) gives us the relation between the degree k and the activity a at a given time t, as $k \propto a^{\frac{1}{1+\beta}}$. Thus, given an activity distribution $F(a)$, we can infer the functional form of the degree distribution $\rho(k)$ by substituting $a \to k^{\frac{1}{1+\beta}}$, finding:

$$\rho(k)dk \propto F(k^{(1+\beta)})k^{\beta}dk. \tag{22}$$

Specifically, by supposing a power-law activity distribution $F(a) \propto a^{-\nu}$ and considering that the degree distribution for a class b is described by Eq. (17), we obtain

$$\rho(k) \propto k^{-[(1+\beta)\nu-\beta]}. \tag{23}$$

where we integrated over time t and reinforcement constant $c(b)$ and we considered the asymptotic regime of large time and activity.

3.2.2 Numerical results

We performed numerical simulations to check the result of Section 3.2.1. We fix the following parameters:

- $N = 10^6$ nodes;
- activity $a \in [\epsilon, 1.0]$ with $\epsilon = 10^{-3}$, power -law distributed so that $F(a) \propto a^{-\nu}$ with $\nu = 2.1$;
- single value of the reinforcement exponent $\beta = \{0.5, 1.0, 1.5, 2.0\}$ and a fixed $c = 1$ for all the nodes;
- $T = 10^5$ evolution steps.

We start with no edge in the system and we draw for each node the activity a_i from the distribution $F(a)$. At each step a randomly chosen node gets active with probability a_i. An active node then connects with probability $p_i(k)$ with a randomly chosen node which have not been yet connected to i or, with probability $1-p_i(k)$, the node calls an already contacted node and no new connection is added to the system. An evolution step corresponds to N of these elementary steps, i.e. for each evolution step we give, on average, the possibility to make a call to every node in the network.

The results are in excellent agreement with the analytical predictions. First, in Fig. 6 we show that the analysis presented in Section 2.1 correctly recovers the reinforcement exponent β_{opt}. Indeed the minimum of the $\chi^2_b(\beta)$ are vertically aligned with the value of β fixed in the simulations.

Then, in Fig. 7 we present the asymptotic growth of the average degree for an activity class (i.e. a collection of nodes bins b featuring similar activity values) and we compare it with the analytical prediction $\langle k(a,t)\rangle \propto (at)^{\frac{1}{1+\beta}}$. In Fig. 8 we show that the shape and the evolution of the $P_i(k,t)$ distribution follows the predicted form of Eq. (17).

The last check regards the overall degree distribution $\rho(k)$ that should follow Eq. (23). In Fig. 9 we compare the activity distribution $F(a) \propto a^{-\nu}$ and the degree distribution $\rho(k)$. The exponent μ leading the $\rho(k) \propto k^{-\mu}$ is in good agreement with the analytically predicted value $\mu = [(1+\beta)\nu - \beta]$.

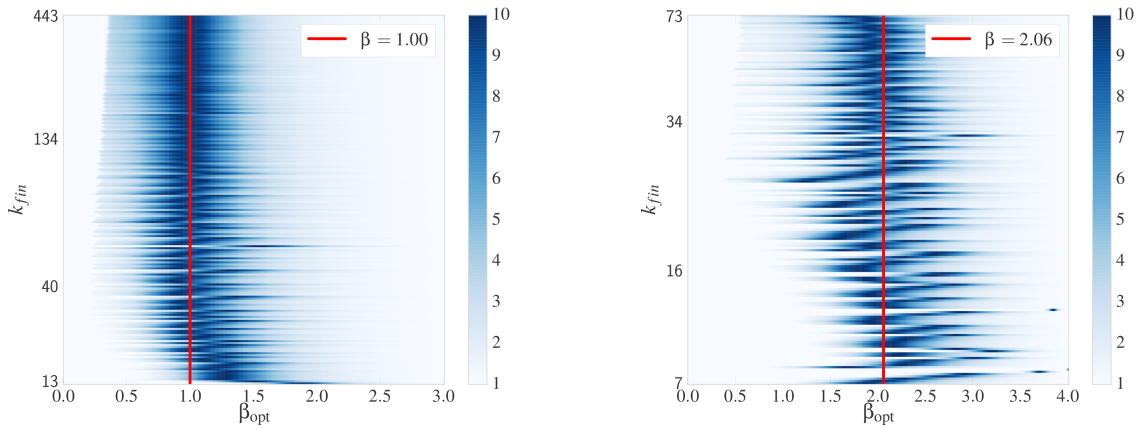

(a) (b)

Fig. 6: The heat-map of $-\ln(\chi^2_b(\beta))$ obtained from the simulation in the same way as in Figure 2 from real data. As one can see the recovered β_{opt} (red vertical line) is in excellent agreement with the value used in the simulation: 1.0 in the (a) panel and 2.06 in the in the (b) panel.

3.2.3 The multi-β case

As shown in Section 2 a single value of the reinforcement exponent β is found to fit most of the $p_b(k)$ curves in both the *APS* and *TMN* datasets, while for *MPN* a single value of β cannot fit all the $p_b(k)$ curves for each activity-degree class b at once. For this reason we further develop the model, letting each node i to feature three parameters: the activity a_i and the reinforcement constant c_i together with the exponent β_i of the underlying reinforcement process.

Since the model with three parameters per node is difficult to handle, we apply some approximations in order to get analytical insight. In particular, we work in simplified single-agent framework, where we focus on a single agent that can only connect to other nodes and never get called. Within this approximation the master equation for the node i reads:

$$P_i(k,t+1) = a_i p(k-1) P_i(k-1,t) + P_i(k,t)\left[a_i(1-p(k)) + (1-a_i)\right]. \tag{24}$$

The continuum limit for large degree k and time t of Eq. (24) is:

$$\frac{\partial P}{\partial t} = -a\left(\frac{c}{k}\right)^{\beta}\left[\frac{\partial P}{\partial k} - \frac{1}{2}\frac{\partial^2 P}{\partial k^2}\right]. \tag{25}$$

The solution for $P_i(k,t)$ is:

$$P_i(k,t) \propto \exp\left[-A\frac{\left(k - C_i t^{\frac{1}{1+\beta_i}}\right)^2}{t^{1/(1+\beta_i)}}\right], \tag{26}$$

where the C_i now reads:

$$C_i = \left[(1+\beta_i)c_i^{\beta} a_i\right]^{\frac{1}{1+\beta_i}}. \tag{27}$$

Again, the average degree $\langle k_i(t)\rangle$ grows as:

$$\langle k_i(t)\rangle \propto C_i t^{\frac{1}{1+\beta_i}}. \tag{28}$$

The result found in Eq. (28) holds for a single class of nodes with a given set of activity a_i and reinforcement constant c_i and strength β_i.

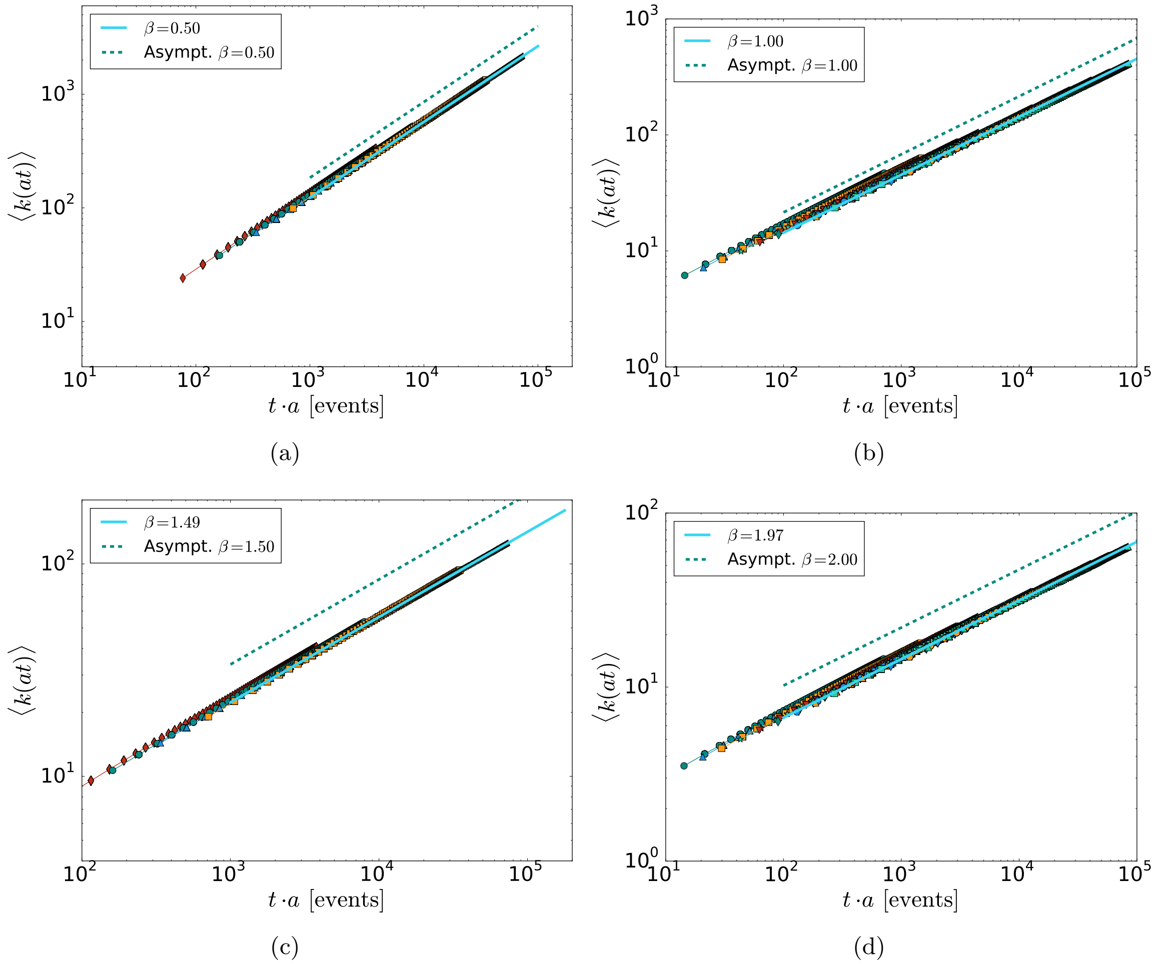

Fig. 7: The average degree $\langle k(at)\rangle$ for different activity classes in the $\beta = 0.5$ (a), $\beta = 1.0$ (b), $\beta = 1.5$ (c) and $\beta = 2.0$ (d) case. The time is rescaled with activity $t \to at$, so that all the curves collapse on a single behavior. We also fit $\langle k(at)\rangle \propto (t/A)^{\frac{1}{1+\beta^*}}$ (cyan solid line) and compare the simulation with the analytical result $\langle k(at)\rangle = A \cdot t^{\frac{1}{1+\beta}}$ (blue dashed line).

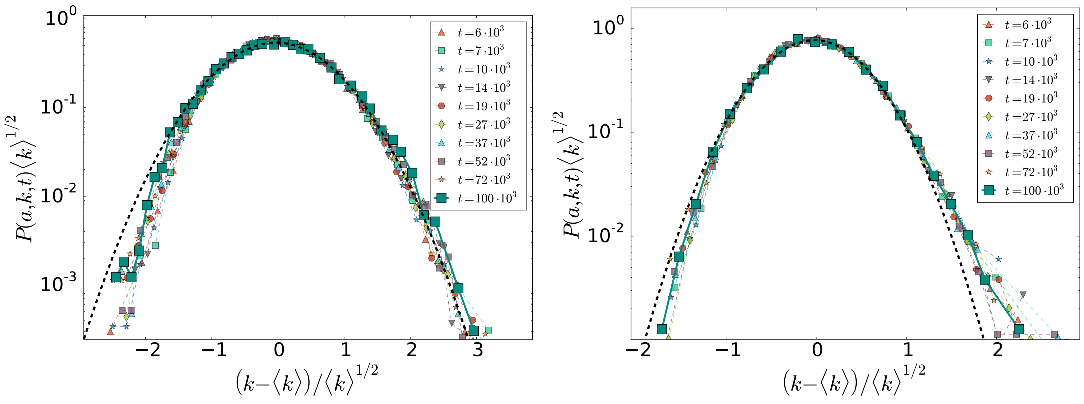

(a) (b)

Fig. 8: The probability distribution $P_a(k,t)$ for a selected activity class a in the simulations with exponent $\beta = 1.0$ (a) and $\beta = 2.0$ (b). We compare different evolution times (see legend) by rescaling the degree $k \to \tilde{k} = (k - \langle k(a,t)\rangle)/\langle k(a,t)\rangle^{1/2}$ on the x-axis and the distribution itself $P_a(k,t) \to \langle k(a,t)\rangle^{1/2} P(a,\tilde{k},t)$ on the y-axis, where $\langle k(a,t)\rangle$ is the average degree at time t for the nodes belonging to the activity class a. We also show the fit of the large time $P(a,k,t)$ with a Gaussian curve (black dashed line) as predicted in Eq. (17).

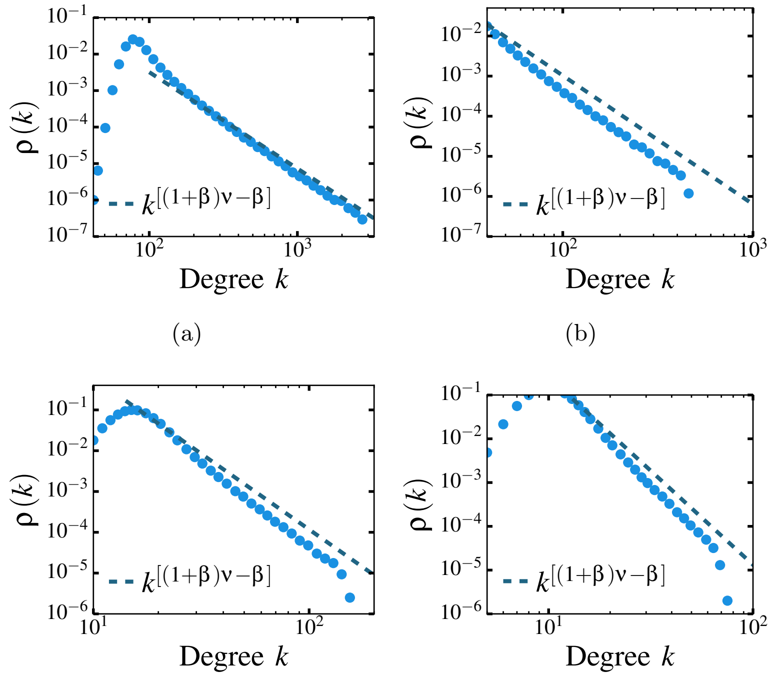

(a) (b)

(c) (d)

Fig. 9: The resulting degree distribution of simulations featuring $\beta = 0.5$ (a), $\beta = 1.0$ (b), $\beta = 1.5$ (c) and $\beta = 1.0$ (d). The analytical predictions (given $F(a) \propto a^{-\nu}$, with $\nu = 2.1$) for the scaling exponent are sown (blue dashed lines).

The average degree $\langle k(a,t)\rangle$ for the activity class a can be computed by integrating over the different values of β_i and c_i:

$$\langle k(a,t)\rangle = \int dc' \int d\beta' \rho(\beta', c'|a) C(a, c', \beta')(t)^{\frac{1}{1+\beta'}} \tag{29}$$

where $\rho(\beta, c|a)$ is the probability for a node of activity a to have a reinforcement exponent and constant equal to β and c. By assuming that the distribution of the exponent β is independent from a and c we can factor out the time-depend term obtaining for the activity class a:

$$\langle k(a,t)\rangle \propto \int d\beta' \rho(\beta') t^{\frac{1}{1+\beta'}}, \tag{30}$$

where $\rho(\beta)$ is the probability distribution of the β parameter.

Let us assume that $\rho(\beta)$ can be written as a sum of Kroenecker δ-functions, i.e.:

$$\rho(\beta) = \frac{1}{\sum_i C_i} \sum_{i=1}^{N_\beta} C_i \delta(\beta - \beta_i). \tag{31}$$

By plugging Eq. (31) in Eq. (30) we find that:

$$\langle k(a,t)\rangle \propto \sum_{i=1}^{N_\beta} C_i t^{\frac{1}{1+\beta_i}} \xrightarrow{t\to\infty} t^{\frac{1}{1+\beta_{\min}}}, \tag{32}$$

so that the minimum value of β_i, $\beta_{\min}$ leads the asymptotic behavior of the $\langle k(a,t)\rangle$ function.

3.2.4 Numerical results

To investigate the multi-β case we performed further numerical simulations considering networks with the following parameters:

- $N = 10^6$ nodes;
- activity $a \in [\epsilon, 1.0]$ with $\epsilon = 10^{-3}$, power -law distributed so that $F(a) \propto a^{-\nu}$ with $\nu = 2.1$;
- (a) reinforcement exponent $\beta = [0.5, 1.5, 2.5]$ with probability $[1/6, 1/3, 1/2]$ (i.e. one sixth of the nodes has $\beta = 0.5$, one third $\beta = 1.5$ and a half of them $\beta = 2.5$ regardless of their activity) and (b) $\beta = [1.0, 1.5, 2.0]$ with equal probability 1/3.
- fixed $c = 1$ for all the nodes;
- $T = 2 \cdot 10^5$ evolution steps.

The numerical procedure is similar to the one described in Section 3.2.2, the difference being that we compute the attachment probability $p_i(k)$ taking into account the reinforcement exponent β_i of the node itself.

In Fig. 10 we show that, in both the cases, we can recover the behavior described in Section 2.1 for real data. In particular Fig. 10(a) (related to the $\beta \in [1, 2]$ case) we observe a clear diagonal pattern of the optimal values of the exponent $\beta(b)$ for the b bins that minimize the $\chi^2_b(\beta)$. In particular $\beta(b)$ varies from $\beta \sim 2.0$ values for lower degree nodes bins up to $\beta \sim 1.0$ values for the larger final degree nodes bins. The figure recalls the situation of the MPC dataset presented in Fig. 2 (b) and in the main paper.

In Fig. 11 we show the asymptotic growth of the average degree $\langle k(a,t)\rangle$ together with the predicted asymptotic behavior proportional to $t^{\frac{1}{1+\beta_{\min}}}$. As one can see, numerical results and the suggested analytical solution are in very good agreement in both the cases.

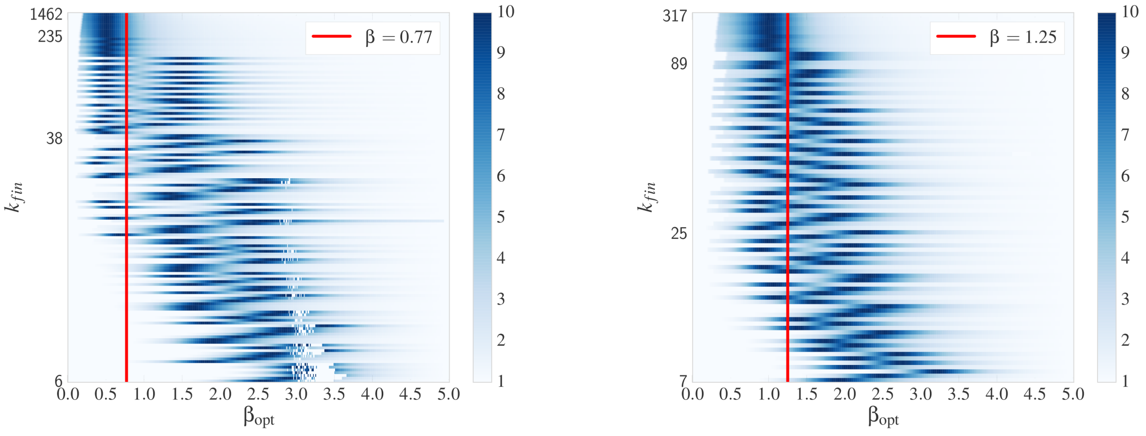

(a) (b)

Fig. 10: The heat-map like matrix of $-\ln(\chi^2(\beta))$ for the simulation with $\beta \in [0.5, 1.5, 2.5]$ (a) and $\beta \in [1.0, 1.5, 2.0]$ (b); the two panels are qualitatively similar to Figure 2 that accounts for real data.

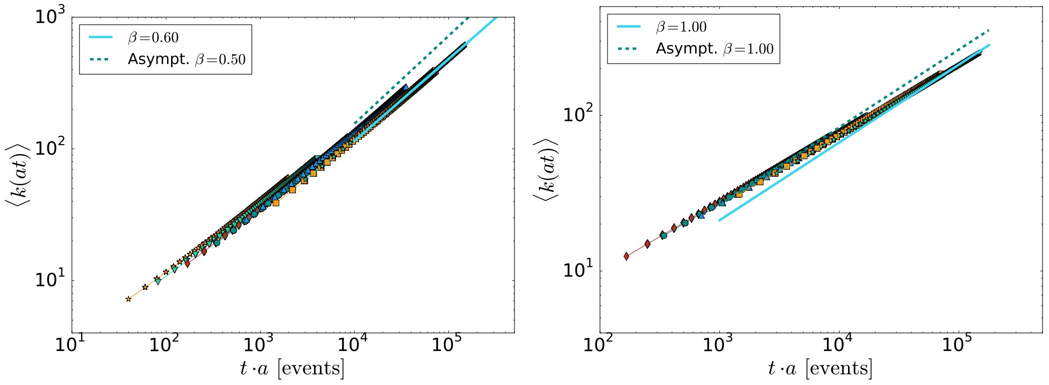

(a) (b)

Fig. 11: The average degree $\langle k(at) \rangle$ for different activity classes in the (a) $\beta \in [0.5, 1.5.2.5]$ and (b) $\beta \in [1.0, 1.5, 2.0]$ case. The time is rescaled with activity $t \to at$, so that all the curves collapse. We also plot the fit $\langle k(at) \rangle \propto (t/A)^{\frac{1}{1+\beta^*}}$ in the long time limit (cyan solid line) and the predicted asymptotic growth $\langle k(at) \rangle = A \cdot t^{\frac{1}{1+\beta_{\min}}}$ (dashed line).

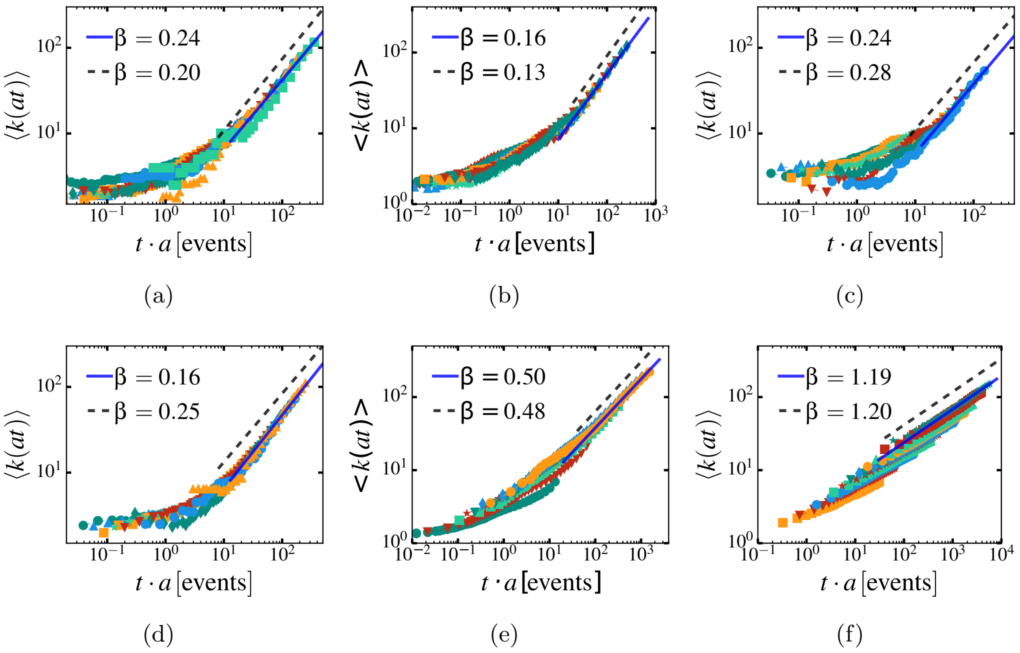

Fig. 12: The average degree $\langle k(at)\rangle$ (each data series corresponds to a different activity class) for: (a) PRA, (b) PRB, (c) PRD, (d) PRE, (e) TWT and (f) MPC. We compare the data for $\langle k(a,t)\rangle$ with the expected behavior (dashed lines) $(at)^{1/(1+\beta_{\rm opt})}$: in panels (a-e) $\beta_{\rm opt}$ has been evaluated according Eq. (6), while in the (f) case we use $\beta_{\rm opt} = \beta_{\rm min} = 1.2$. We also plot the power-law fit $\langle k(a,t)\rangle \propto (at)^{1/(1+\beta^*)}$ (solid lines) for comparison.

3.3 Comparison with real data

In Fig. 12 we present the comparison between prediction and real data for the PRA, PRB, PRD, PRE, TMN and MPN datasets. The $\langle k(a,t)\rangle$ curve of each activity class is shown with the time rescaled with the activity of each activity class, i.e. $t \to at$. In the MPC case we use as $\beta_{\rm opt} = \beta_{\rm min} = 1.2$ (the β value found in the largest degree bins of Fig. 2 (b)). In all the other cases, as the $\beta_{\rm opt}$ fits correctly most of the curves, we use the $\beta_{\rm opt}$ value returned by our analysis.

Finally, in Fig. 13 we present the degree distributions, together with the predicted functional form of degree distribution as found in Table (1) in the main paper.

References

[1] Radicchi, F., Fortunato, S., Markines, B., and Vespignani, A. *Phys. Rev. E* **80**, 056103 Nov (2009).

[2] Clauset, A., Shalizi, C. R., and Newman, M. E. J. *SIAM Review* **51**(4), 661–703 (2009).

[3] Perra, N., Goncalves, B., Pastor-Satorras, R., and Vespignani, A. *Sci. Rep.* **2** 06 (2012).

[4] Starnini, M. and Pastor-Satorras, R. *Phys. Rev. E* **87**, 062807 Jun (2013).

[5] Karsai, M., Perra, N., and Vespignani, A. *Sci. Rep.* **4**, 4001 02 (2014).

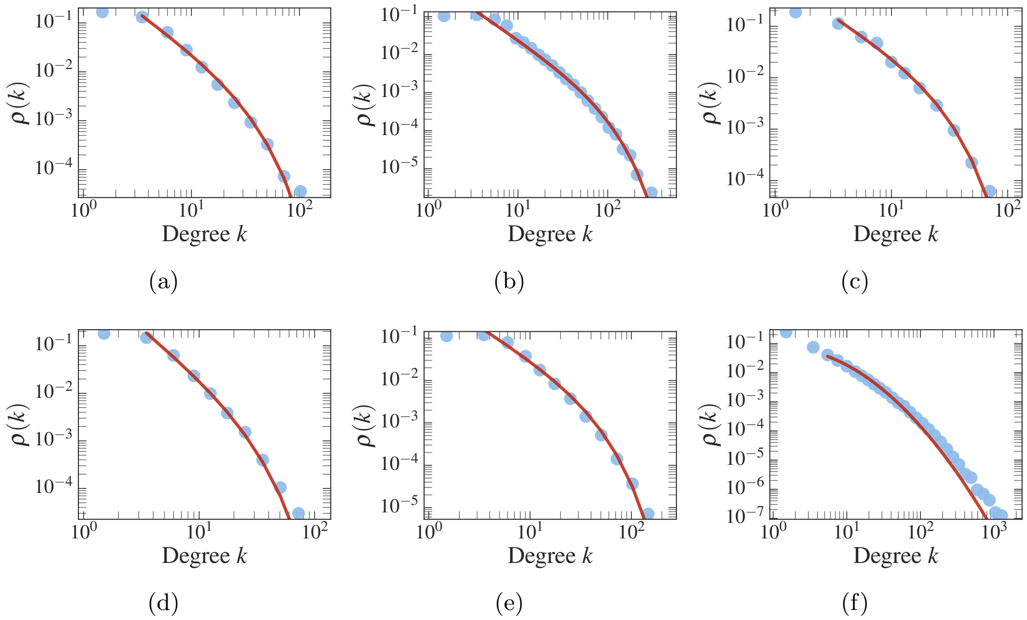

Fig. 13: The degree distribution $\rho(k)$ for: (a) PRA, (b) PRB, (c) PRD, (d) PRE, (e) PRL and (f) TMN (blue circles). We compare the results with the predicted behavior of Table (1) of main paper given the parameters of Table (1) (red solid lines). We use the single value of β_{opt} defined by Eq. (6) in all the cases. As in Fig. 1 we show the data and fit starting from the lower bound of the distribution, including all the statistically significant data in the plot.
